# Phenotyping left ventricular systolic dysfunction in asymptomatic individuals for improved risk stratification

**DOI:** 10.1093/ehjci/jead218

**Published:** 2023-09-12

**Authors:** Elisa Rauseo, Musa Abdulkareem, Abbas Khan, Jackie Cooper, Aaron M Lee, Nay Aung, Gregory G Slabaugh, Steffen E Petersen

**Affiliations:** William Harvey Research Institute, NIHR Barts Biomedical Research Centre, Queen Mary University London, Charterhouse Square, London EC1M 6BQ, UK; Barts Heart Centre, St Bartholomew’s Hospital, Barts Health NHS Trust, West Smithfield, London EC1A 7BE, UK; William Harvey Research Institute, NIHR Barts Biomedical Research Centre, Queen Mary University London, Charterhouse Square, London EC1M 6BQ, UK; Barts Heart Centre, St Bartholomew’s Hospital, Barts Health NHS Trust, West Smithfield, London EC1A 7BE, UK; Health Data Research UK, 215 Euston Rd, London NW1 2BE, UK; School of Electronic Engineering and Computer Science, Queen Mary University of London, UK; Digital Environment Research Institute, Queen Mary University of London, UK; William Harvey Research Institute, NIHR Barts Biomedical Research Centre, Queen Mary University London, Charterhouse Square, London EC1M 6BQ, UK; William Harvey Research Institute, NIHR Barts Biomedical Research Centre, Queen Mary University London, Charterhouse Square, London EC1M 6BQ, UK; William Harvey Research Institute, NIHR Barts Biomedical Research Centre, Queen Mary University London, Charterhouse Square, London EC1M 6BQ, UK; Barts Heart Centre, St Bartholomew’s Hospital, Barts Health NHS Trust, West Smithfield, London EC1A 7BE, UK; School of Electronic Engineering and Computer Science, Queen Mary University of London, UK; Digital Environment Research Institute, Queen Mary University of London, UK; Alan Turing Institute, British Library, 96 Euston Rd, London NW1 2DB, UK; William Harvey Research Institute, NIHR Barts Biomedical Research Centre, Queen Mary University London, Charterhouse Square, London EC1M 6BQ, UK; Barts Heart Centre, St Bartholomew’s Hospital, Barts Health NHS Trust, West Smithfield, London EC1A 7BE, UK; Health Data Research UK, 215 Euston Rd, London NW1 2BE, UK; Alan Turing Institute, British Library, 96 Euston Rd, London NW1 2DB, UK

**Keywords:** left ventricular systolic dysfunction, risk stratification, prognosis, cardiovascular events, heart failure, cardiovascular magnetic resonance

## Abstract

**Aims:**

Left ventricular systolic dysfunction (LSVD) is a heterogeneous condition with several factors influencing prognosis. Better phenotyping of asymptomatic individuals can inform preventative strategies. This study aims to explore the clinical phenotypes of LVSD in initially asymptomatic subjects and their association with clinical outcomes and cardiovascular abnormalities through multi-dimensional data clustering.

**Methods and results:**

Clustering analysis was performed on 60 clinically available variables from 1563 UK Biobank participants without pre-existing heart failure (HF) and with left ventricular ejection fraction (LVEF) < 50% on cardiovascular magnetic resonance (CMR) assessment. Risks of developing HF, other cardiovascular events, death, and a composite of major adverse cardiovascular events (MACE) associated with clusters were investigated. Cardiovascular imaging characteristics, not included in the clustering analysis, were also evaluated. Three distinct clusters were identified, differing considerably in lifestyle habits, cardiovascular risk factors, electrocardiographic parameters, and cardiometabolic profiles. A stepwise increase in risk profile was observed from Cluster 1 to Cluster 3, independent of traditional risk factors and LVEF. Compared with Cluster 1, the lowest risk subset, the risk of MACE ranged from 1.42 [95% confidence interval (CI): 1.03–1.96; *P* < 0.05] for Cluster 2 to 1.72 (95% CI: 1.36–2.35; *P* < 0.001) for Cluster 3. Cluster 3, the highest risk profile, had features of adverse cardiovascular imaging with the greatest LV re-modelling, myocardial dysfunction, and decrease in arterial compliance.

**Conclusions:**

Clustering of clinical variables identified three distinct risk profiles and clinical trajectories of LVSD amongst initially asymptomatic subjects. Improved characterization may facilitate tailored interventions based on the LVSD sub-type and improve clinical outcomes.

## Graphical Abstract

**Graphical Abstract:**
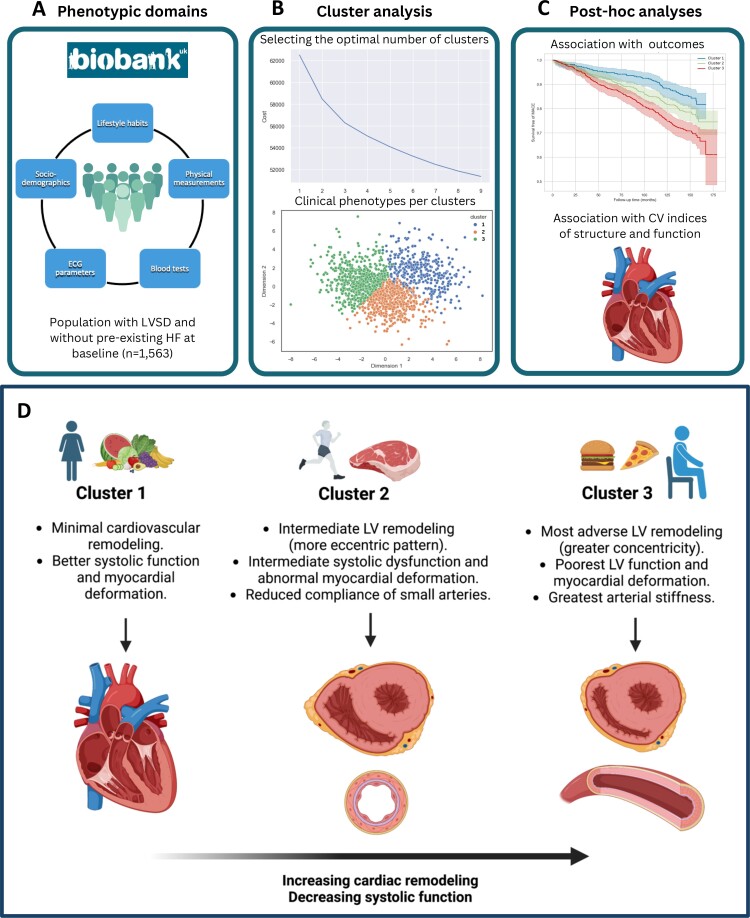
Using clustering analysis of clinical data, we identified three distinct LVSD phenotypes amongst initially asymptomatic subjects (*A*, *B*). These phenotypes correlated with varying risks of adverse CV events (*C*) and were characterized by unique clinical features and CV imaging patterns (*D*). CV, cardiovascular; LVSD, left ventricular systolic dysfunction.

## Introduction

Asymptomatic left ventricular systolic dysfunction (LVSD) represents a preclinical stage of heart failure (HF) characterized by cardiac structural and functional abnormalities.^1^ Despite the absence of symptoms, LVSD can progress to symptomatic HF with increased cardiovascular (CV) morbidity and mortality,^2^ emphasizing the importance of early detection and targeted interventions.

Data available on prevalence and prognosis of LVSD with or without symptoms in the general population are somehow limited mainly due to the lack of consensus on how best to identify such individuals.^3^ Reliance on left ventricular ejection fraction (LVEF) as a single measure of ventricular function has led to varied cut-offs and a heterogeneous group of individuals with different clinical phenotypes and risks. Given the heterogeneity and the association with poor outcomes even in the absence of clinical CV diseases,^2^ comprehensive evaluation of available clinical data may identify asymptomatic individuals at higher risk of LVSD progression and guide more aggressive preventive strategies.

Clustering, an unsupervised machine learning technique, can reveal hidden patterns and homogeneous groups within a heterogeneous population. Its application in various CV settings has enhanced disease characterization,^4,5^ providing insights into pathophysiology, diagnosis, and treatment.

To address limitations in prognostic data for LVSD, we employed cluster analysis of clinically available data to explore risk profiles. Our hypothesis was that a data-driven approach could identify distinct clinical phenotypes in initially asymptomatic individuals associated with different levels of CV impairments, adverse outcomes, and clinical trajectories.

## Methods

### UK Biobank data set

The UK Biobank is a large prospective population study that collects extensive phenotypic and genotypic information from over 500 000 participants recruited across the UK.^6^ Baseline data include socio-demographics, lifestyle factors, medical history, genetics, physical measures, and health-related outcomes, with longitudinal follow-up. Imaging studies, including cardiovascular magnetic resonance (CMR), were conducted on more than 48 000 participants.

This study adheres to the principles outlined in the Declaration of Helsinki and has received ethical approval from the NHS National Research Ethics Service on 17 June 2011 (Ref 11/NW/0382), with an extension on 18 June 2021 (Ref 21/NW/0157). Written informed consent was obtained from all participants.

### Study population

Participants without clinically overt HF, cardiomyopathy, and dyspnoea on exertion at baseline (see Supplementary data online, *Table S1*), and with evidence of LVSD at the subsequent CMR assessment, were selected as our study population (*n* = 1563). LVSD was defined as LVEF < 50% according to previously published data.^2^

### Phenotypic domains

Baseline characteristics, including socio-demographics, lifestyle factors, physical characteristics, electrocardiogram (ECG) parameters, and laboratory data, served as phenotypic domains for clustering analysis. These variables were selected as they represent important CV health metrics^7^ and contribute to risk assessment. Detailed descriptions of the phenotypic features are provided in Supplementary data online, *Table S2* and *Methods S1*.

### Imaging parameters

CMR-derived features, not included in the clustering analysis, were assessed to provide additional evaluation of the clusters. A detailed description of CMR image acquisition and analysis is included in Supplementary data online, *Methods S2*. Briefly, CMR images were acquired using 1.5 Tesla scanners (MAGNETOM Aera, Syngo Platform VD13A, Siemens Healthcare, Erlangen, Germany) according to a pre-defined acquisition protocol.^8^ Image analysis was performed using Circle Cardiovascular Imaging 42 post-processing software (Version 5.11, Circle Cardiovascular Imaging Inc., Calgary, Canada).

These features included left ventricular (LV) and right ventricular (RV) volumes and their stroke volumes, LV mass and LV mass-to-volume (M/V) ratio, LV and RV ejection fraction, indices of myocardial performance such as LV global function index (LVGFI), and left ventricular strain [global longitudinal strain (GLS), global circumferential strain (GCS), global radial strain (GRS), and torsion].^9,10^

Additionally, markers of arterial function, including total arterial compliance (TAC), aortic distensibility (AoD), and systemic vascular resistance (SVR) were analysed to estimate ventricular–arterial coupling and assess changes in LVSD.^11,9^

### Ascertainment of outcomes

Clinical outcomes occurring after the baseline (incident events) were determined using selected UK Biobank fields with recorded dates for censoring (see Supplementary data online, *Table S3*). Survival analyses involved censoring individuals based on the event date, date of death, or end of follow-up (26 March 2021), whichever came first.

The clinical endpoints of interest were all-cause HF, all-cause mortality, and other CV events. All-cause HF encompassed HF, pulmonary oedema, or any cardiomyopathy as a plausible underlying cause.^12^ Incident of other CV events included non-fatal myocardial infarction, cardiac arrhythmia, stroke, or peripheral artery disease. Additionally, a composite endpoint of major adverse cardiovascular events (MACE), comprising HF, other CV events, or CV death, was assessed.

### Statistical analysis

Clustering analysis identified patterns in phenotypic features by grouping unlabelled data into similar characteristic-based clusters. A more detailed description of the statistical analysis, including clustering, is provided in Supplementary data online, *Methods S3*.

Briefly, we selected 60 clinical features covering all phenotypic domains after removing variables with >30% missingness and highly correlated features (see Supplementary data online, *Table S2*). Clustering analysis utilized the k-prototype algorithm^13^ due to its efficiency in handling large data sets with mixed variable types.^14^ The stability of clustering results was assessed using re-sampling-based methods to account for sampling variability.^15^ Clustering was performed blindly to clinical outcomes and CMR metrics, which served for clinical validation.

The association between cluster membership and clinical outcomes was assessed using unadjusted and multivariate Cox proportional hazards models, ensuring no violations were present. Adjusted models included traditional CV risk factors (hypertension, diabetes, and previous myocardial infarction) and LVEF values as covariates. Interaction terms in Cox regression models were used to test differential associations between cluster membership and LVEF with clinical outcomes.

All analyses were conducted using Python 3.8.10 (Python Software Foundation, Delaware USA) and Scikit-learn version 0.23.2.^16^ A two-sided *P* < 0.05 was considered statistically significant for all analyses.

## Results

### Study population characteristics

The LVSD cohort (*n* = 1563) primarily consisted of middle-aged Caucasian males (76%), with a high prevalence of CV risk factors (hypertension 29.4%, hypercholesterolaemia 20.1%, and diabetes 5.3%) and a median LVEF of 47.6% (44.7–49.1%). Prevalent co-morbidities, including previous myocardial infarction, were infrequent (2.6%) (see Supplementary data online, *Table S4*). Over an average follow-up of 11.4 years (maximum 15 years), 215 participants developed clinical HF, 336 experienced other CV events, and 36 died from various causes. The overall incidence of MACE was 23.2% (see Supplementary data online, *Table S5*).

### Identifying distinct clusters

Cluster analysis using the k-prototypes algorithm grouped LVSD participants based on 60 phenotypic features. The elbow method determined that a three-cluster model best fit the data set (see Supplementary data online, *Figure S3*). This model produced clinically interpretable patterns (*Tables 1* and *2* and Supplementary data online, *Table S6*).

**Table 1:** Clinical characteristics by clusters

	Cluster 1 (*n* = 348)	Cluster 2 (*n* = 596)	Cluster 3 (*n* = 619)	*P*-value	Cluster 1 vs. Cluster 2	Cluster 1 vs. Cluster 3	Cluster 2 vs. Cluster 3
Socio-demographics							
Age, years	55.96 ± 7.39	55.93 ± 7.97	56.71 ± 7.23	0.24	0.95	0.12	0.07
Female, *n* (%)	323 (92.8)	6 (1.01)	35 (5.65)	**<0**.**001**	**<0**.**001**	**<0**.**001**	**<0**.**001**
Ethnicity, *n* (%)				0.50	0.32	0.45	0.50
White	336 (96.5)	582 (97.82)	600 (97.4)				
Other ethnicities	12 (3.44)	13 (2.19)	16 (2.59)				
Townsend deprivation index	2.4 (3.7–0.2)	2.6 (3.8–0.5)	2.8 (3.9–0.6)	0.09	0.11	**0**.**04**	0.50
Educational level, *n* (%)				0.35	0.63	0.39	0.17
High	188 (54)	334 (56)	314 (50.72)				
Intermediate	113 (32.47)	176 (29.53)	202 (32.63)				
Low	47 (13.5)	86 (14.42)	103 (16.63)				
Co-morbidities, *n* (%)							
Previous myocardial infarction	4 (1.15)	17 (2.85)	19 (3.07)	0.16	0.14	0.09	0.96
Stroke	4 (1.15)	5 (0.84)	11 (1.78)	0.33	0.89	0.63	0.24
Chronic obstructive pulmonary disease	5 (1.44)	3 (0.50)	8 (1.29)	0.26	0.25	1	0.25
Asthma	52 (14.94)	71 (11.91)	96 (15.50)	0.16	0.22	0.88	0.08
Atrial fibrillation	1 (0.29)	25 (4.19)	23 (3.72)	**0**.**002**	**0**.**004**	**0**.**002**	0.78
Peripheral artery disease	4 (1.15)	8 (1.34)	1 (0.16)	0.06	1	0.11	**0**.**04**
Hypertension	79(22.70)	123(20.65)	258(41.68)	**<0**.**001**	0.51	**<0**.**001**	**<0**.**001**
Diabetes	13 (3.74)	9 (1.51)	61 (9.85)	**<0**.**001**	0.05	**<0**.**001**	**<0**.**001**
Hypercholesterolaemia	38 (10.92)	96 (16.11)	178 (28.76)	**<0**.**001**	**0**.**03**	**<0**.**001**	**<0**.**001**
Chronic kidney disease	3 (0.86)	3 (0.50)	8 (1.29)	0.34	0.81	0.77	0.25
Physical measurements							
Waist hip ratio	0.8 ± 0.07	0.89 ± 0.05	0.95 ± 0.06	**<0**.**001**	**<0**.**001**	**<0**.**001**	**<0**.**001**
Fat mass index, kg/m^2^	8.6 (6.9–10.9)	5.5 (4.5–6.7)	8 (6.7–9.9)	**<0**.**001**	**<0**.**001**	**0**.**003**	**<0**.**001**
Fat free mass index, kg/m^2^	16.7 ± 1.5	19.87 ± 1.54	21.05 ± 1.88	**<0**.**001**	**<0**.**001**	**<0**.**001**	**<0**.**001**
DBP, mmHg	81.7 ± 10.3	80.78 ± 8.66	87.95 ± 9.34	**<0**.**001**	0.20	**<0**.**001**	**<0**.**001**
SBP, mmHg	135.2 ± 17.2	133.84 ± 14.61	143.58 ± 15.62	**<0**.**001**	0.13	**<0**.**001**	**<0**.**001**
Pulse rate, bpm	70.9 ± 10.3	63.82 ± 9.49	72.9 ± 11.23	**<0**.**001**	**<0**.**001**	**0**.**006**	**<0**.**001**

Values: mean (±SD) for continuous variables and number (percentage) for categorical variables.

Data presented as median (interquartile range) when absolute skew is ≥0.9. *P*-values indicate variable comparisons across clusters, with significance denoted by bold values (*P* < 0.05).

DBP, diastolic blood pressure; SBP, systolic blood pressure.

**Table 2:** Lifestyle behaviours by clusters

	Cluster 1 (*n* = 348)	Cluster 2 (*n* = 596)	Cluster 3 (*n* = 619)	*P*-value	Cluster 1 vs. Cluster 2	Cluster 1 vs. Cluster 3	Cluster 2 vs. Cluster 3
Lifestyle habits							
Smoking history	139 (39.94)	233 (39.09)	327 (52.82)	**<0**.**001**	0.85	**<0**.**001**	**<0**.**001**
Alcohol intake, frequency				**<0**.**001**	**<0**.**001**	**0**.**005**	0.13
Never	26 (7.47)	18 (3.02)	28 (4.52)				
Special occasion only	35 (10)	26 (4.36)	45 (7.26)				
1–3 times a month	45 (12.9)	41 (6.87)	46 (7.43)				
1–2 times a week	77 (22.1)	142 (23.82)	153 (24.71)				
3–4 times a week	84 (24.13)	173 (29.02)	169 (27.3)				
Daily or almost daily	81 (23.27)	196 (32.88)	178 (28.76)				
Time watching TV, h/day	2.33 ± 1.47	2.11 ± 1.3	2.76 ± 1.47	**<0**.**001**	**0**.**02**	**<0**.**001**	**<0**.**001**
Time using computer h/day	0.95 ± 1.06	1.46 ± 1.6	1.53 ± 1.43	**<0**.**001**	**<0**.**001**	**<0**.**001**	0.42
Sleep duration, h/day	7.22 ± 0.99	7.05 ± 0.91	7.24 ± 1.05	**<0**.**001**	**0**.**007**	0.74	**<0**.**001**
Physical activity, IPAQ score	1523 (711–2995)	2161 (1108–3830)	1371 (575–2655)	**<0**.**001**	**<0**.**001**	0.1	**<0**.**001**
Dietary habits							
Fruits intake, servings per day				**<0**.**001**	0.09	**<0**.**001**	**0**.**01**
<3 servings a day	174 (50)	348 (58.38)	401 (64.78)				
3–4 servings a day	74 (21.26)	108 (18.12)	118 (19.06)				
4–6 servings a day	75 (21.55)	103 (17.28)	77 (12.43)				
≥6 servings a day	25 (7.18)	37 (6.20)	23 (3.72)				
Vegetables intake, servings per day				0.79	0.88	0.42	0.71
<3 servings a day	241 (69.25)	421 (70.63)	455 (73.5)				
3–4 servings a day	60 (17.24)	105 (17.61)	101 (16.31)				
4–6 servings a day	36 (10.34)	54 (9.06)	48 (7.75)				
≥6 servings a day	11 (3.16)	16 (2.68)	15 (2.42)				
Oily fish intake, frequency				0.09	0.05	0.85	0.06
0–1 time/week	40 (11.49)	45 (7.55)	64 (10.3)				
1–2 times/week	253 (72.7)	432 (72.48)	457 (73.82)				
≥2 times/week	55 (15.8)	119 (19.96)	98 (15.83)				
Non-oily fish intake, frequency				**0**.**02**	**0**.**01**	**0**.**03**	0.61
0–1 time/week	27 (7.75)	20 (3.35)	24 (3.87)				
1–2 times/week	274 (75.86)	470 (78.85)	497 (80.29)				
≥2 times/week	57 (16.37)	106 (17.78)	98 (15.83)				
Processed food intake, frequency				**<0**.**001**	**<0**.**001**	**<0**.**001**	**<0**.**001**
0–1 time/week	41 (11.78)	53 (8.89)	26 (4.2)				
1–2 times/week	236 (77.8)	329 (55.20)	312 (50.4)				
≥2 times/week	71 (20.4)	214 (35.90)	281 (45.39)				
Red meat intake, frequency				**<0**.**001**	0.8	**<0**.**001**	**<0**.**001**
0–1 time/week	46 (13.21)	66 (11.07)	36 (5.81)				
1–2 times/week	261 (75)	457 (76.67)	473 (76.41)				
2–3 times/week	3 (0.86)	6 (1.01)	13 (2.1)				
≥4 times/week	38 (10.92)	67 (11.24)	97 (15.67)				
Coffee intake, cups per day				**0**.**003**	0.06	**<0**.**001**	0.15
0–1 cup/day	174 (50)	255 (42.78)	240 (38.77)				
1–2 cups/day	72 (20.68)	126 (21.14)	122 (19.7)				
≥3 cups/day	102 (29.31)	215 (36.07)	257 (41.51)				
Tea intake, cups per day				**0**.**02**	0.21	0.53	**0**.**004**
0–1 cup/day	93 (26.72)	135 (22.65)	186 (30.04)				
1–3 cups/day	56 (16.09)	86 (14.42)	99 (15.99)				
≥3 cups/day	199 (57.18)	375 (62.91)	334 (53.95)				

The *P* value indicates comparisons of variables across clusters and bold values indicate statistical significance (*P* < 0.05).

Cluster 1 (*n* = 348) consisted mainly of females with the lowest proportions of atrial fibrillation and hypercholesterolaemia. They exhibited the healthiest diet habits, consuming the least alcohol and processed food. Metabolically, they had a favourable profile with high-density lipoprotein (HDL) and low triglyceride levels. Their ECG showed the shortest *P* and QRS wave durations.

Cluster 2 (*n* = 596) comprised mostly males with a higher burden of hypercholesterolaemia and atrial fibrillation. They had higher alcohol and processed food intake compared to Cluster 1. This group displayed the lowest sedentary behaviours, lowest adiposity index, lowest blood pressure values, lowest pulse rate, longest QRS duration, and lowest ventricular rate on ECG.

Cluster 3 (*n* = 619) was predominantly males with the highest burden of CV risk factors, including diabetes, hypertension, and hypercholesterolaemia. They were the least active, had the unhealthiest diet habits, and had the highest indices of adiposity. Physical examination revealed higher pulse rate and blood pressure values. Blood tests showed a worse lipid profile, higher urinary creatinine, glycate, and C-reactive protein (CRP) levels compared to the other clusters.

### Comparing imaging characteristics

CMR analysis showed significant differences across the clusters (*Table 3*). Cluster 1 had the smallest RV and LV volumes, better bi-ventricular systolic function, and smallest LV mass and M/V ratio. These subjects tended to have better LV deformation by strain measurements (more negative GLS and GCS values and higher GRS values) and had the lowest AoD and peripheral vascular resistance (SVR).

**Table 3:** Cardiovascular imaging characteristics by clinical phenotypes

	Cluster 1 (*n* = 348)	Cluster 2 (*n* = 596)	Cluster 3 (*n* = 619)	*P*-value	Cluster 1 vs. Cluster 2	Cluster 1 vs. Cluster 3	Cluster 2 vs. Cluster 3
CMR indices of cardiac structure and function							
LVEDVi (mL/m^2^)	80.77 ± 16.32	93.03 ± 18.25	84.38 ± 18.11	**<0**.**001**	**<0**.**001**	**0**.**002**	**<0**.**001**
LVESVi (mL/m^2^)	41.87 (36.59–47.93)	47.91 (43.09–54.76)	43.95 (38.95–50.91)	**<0**.**001**	**<0**.**001**	**<0**.**001**	**<0**.**001**
LVSVi (mL/m^2^)	37.43 ± 7.14	42.93 ± 8.24	38.16 ± 8.19	**<0**.**001**	**<0**.**001**	0.15	**<0**.**001**
LVMi (g/m^2^)	43.65 (39.11–50.25)	52.22 (47.92–58.24)	52.50 (46.72–58.82)	**<0**.**001**	**<0**.**001**	**<0**.**001**	0.71
LVEF (%)	48 (45.43–49.21)	47.6 (45.03–49.09)	47.2 (44.23–48.91)	**0**.**003**	0.14	**0**.**001**	**0**.**002**
RVEDVi (mL/m^2^)	76.94 ± 13.69	94.39 ± 15.65	83.03 ± 15.69	**<0**.**001**	**<0**.**001**	**<0**.**001**	**<0**.**001**
RVESVi (mL/m^2^)	37.74 ± 8.68	48.89 ± 9.62	42.84 ± 10.15	**<0**.**001**	**<0**.**001**	**<0**.**001**	**<0**.**001**
RVSVi (mL/m^2^)	39.19 ± 8.43	45.50 ± 9.57	40.54 ± 9.57	**<0**.**001**	**<0**.**001**	**0**.**02**	**<0**.**001**
RVEF (%)	50.96 ± 7.32	48.12 ± 6.17	48.81 ± 7.47	**<0**.**001**	**<0**.**001**	**<0**.**001**	0.08
M/V ratio (g/mL)	0.56 (0.51–0.61)	0.58 (0.53–0.63)	0.63 (0.57–0.69)	**<0**.**001**	**0**.**001**	**<0**.**001**	**<0**.**001**
LVGFI (%)	36.64 (34.05–38.68)	35.86 (33.37–37.72)	33.97 (31.21–36.13)	**<0**.**001**	**<0**.**001**	**<0**.**001**	**<0**.**001**
CMR indices of arterial function							
AoD (×10^−3^/mmHg)	0.84 (0.52–1.55)	1.37 (0.81–2.14)	1.11 (0.66–1.71)	**<0**.**001**	**<0**.**001**	**<0**.**001**	**<0**.**001**
Indexed SVR (mmHg.min./L.m^2^)	22.81 (18.56–27.56)	32.06 (27.12–38.53)	31.28 (25.40–36.98)	**<0**.**001**	**<0**.**001**	**<0**.**001**	**0**.**02**
Indexed TAC (mL/mmHg/m^2^)	0.66 (0.53–0.86)	0.70 (0.56–0.90)	0.60 (0.49–0.78)	**<0**.**001**	0.12	**<0**.**001**	**<0**.**001**
CMR indices of cardiac mechanics							
GLS (%)	−15.31 (16.67–13.41)	−15.19 (16.56–13.53)	−14.11 (15.69–12.10)	**<0**.**001**	0.92	**<0**.**001**	**<0**.**001**
GCS (%)	−14.63 (15.91–13.05)	−14.75 (15.83–13.38)	−14.19 (15.33–12.57)	**<0**.**001**	0.94	**<0**.**001**	**<0**.**001**
GRS (%)	21.28 ± 4.82	21.08 ± 4.47	19.78 ± 5.14	**<0**.**001**	0.65	**<0**.**001**	**0**.**001**
Torsion (degrees)	0.70 ± 0.53	0.68 ± 0.46	0.74 ± 0.47	0.17	0.64	0.29	0.05

The *P* value indicates comparisons of variables across clusters and bold values indicate statistical significance (*P* < 0.05).

AoD, aortic distensibility; GCS, global circumferential strain; GLS, global longitudinal strain; GRS, global radial strain; LVEDVI, left ventricular end-diastolic volume index; LVEF, left ventricular ejection fraction; LVMI, left ventricular mass index; LVESVI, left ventricular end-systolic volume index; LVGFI, left ventricle global function index; LVSV, left ventricular stroke volume index; M/V, LV mass-to-volume ratio; RVEDVI, right ventricular end-diastolic volume index; RVEF, right ventricular ejection fraction; RVESVI, right ventricular end-systolic volume index; RVSVI, right ventricular stroke volume index; SVR, systemic vascular resistance; TAC, total arterial compliance.

Cluster 2 had the largest RV and LV volumes, lowest RVEF, significantly lower global LV performance and marginally lower LVEF than Cluster 1. These subjects had also increased LV mass and M/V ratio compared to Cluster 1, the least stiffening of conduct arteries (highest TAC and AoD), and the highest peripheral vascular resistance.

Cluster 3 had smaller ventricular volumes than Cluster 2 but had the lowest LVEF and LV performance, highest M/V ratio, worst LV deformation by strain metrics, and highest arterial load (lowest TAC).

### Cluster associations with clinical outcomes

**Figure 1:**
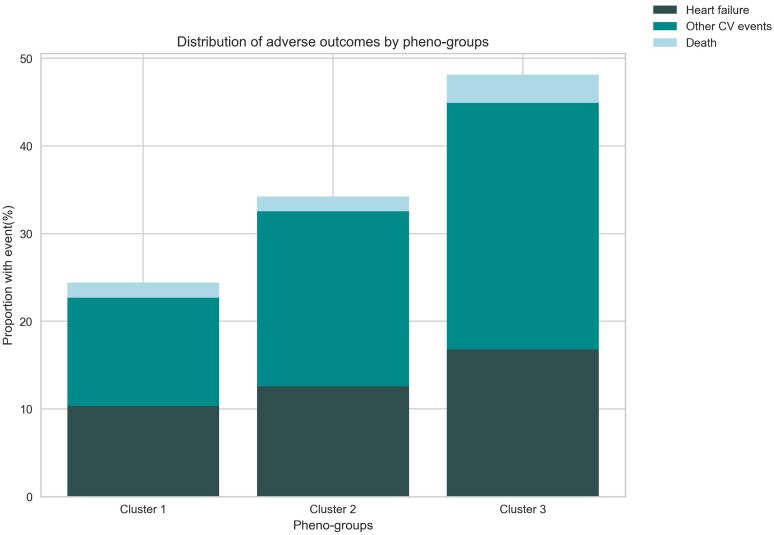
Distribution of outcomes by phenogroups. CV, cardiovascular.

**Figure 2:**
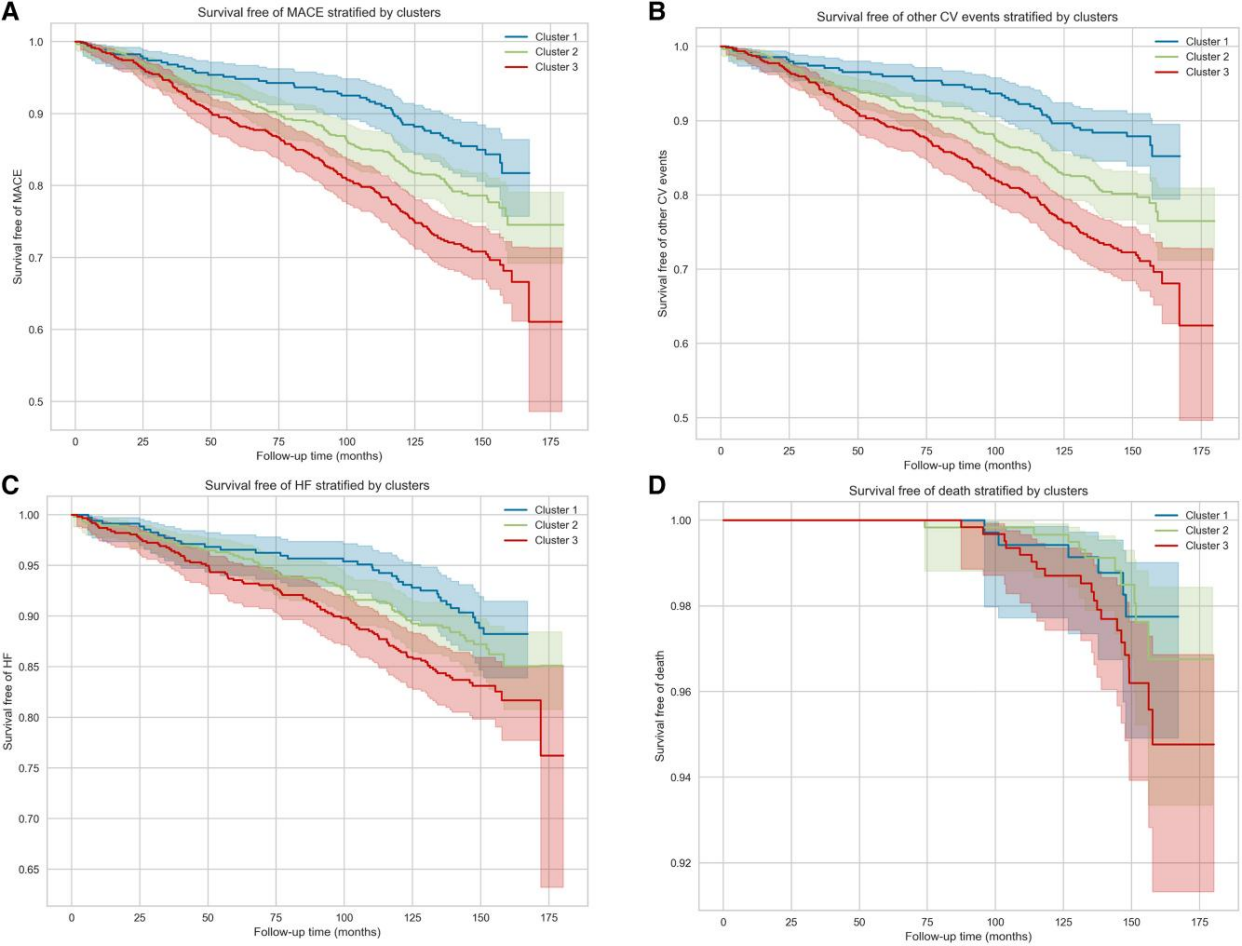
Survival free of MACE, other CV events, HF, and all-cause mortality by cluster. Kaplan–Meier curves for MACE (*A*), other CV events (*B*), HF (*C*), and all-cause death (*D*) stratified by cluster. CV, cardiovascular; HF, heart failure; MACE, major adverse cardiovascular events.

Overall, incident CV events, including HF and other CV events, varied significantly by clusters (*Table 4*), with a stepwise increase in risk from Cluster 1 (lowest risk) to Cluster 3 (highest risk) (*Figure 1*). A similar pattern was observed for all-cause death, albeit not statistically significant. Kaplan–Meier analysis confirmed significantly different clinical trajectories for CV outcomes (HF, other CV events, and combined MACE) across the clusters (*Figure 2*).

**Table 4:** Clusters and adverse outcomes on Cox proportional hazards analysis

	Cluster 1 (*n* = 348)	Cluster 2 (*n* = 596)	Cluster 3 (*n* = 619)	*P*-value	Cluster 2: LVEF *P*-value	Cluster 3: LVEF *P*-value
Outcome, *n* (%)						
Incident HF	36 (10.34)	75 (12.58)	104 (16.8)	0.011		
Other CV events	43 (12.36)	119 (19.97)	174 (28.11)	<0.001		
Death for all causes	6 (1.72)	10 (1.68)	20 (3.23)	0.14		
Combined MACE	53 (15.23)	128 (21.48)	183 (29.56)	<0.001		
Unadjusted, HR (95% CI)						
Incident HF	1	1.26 (0.84–1.87)	1.70 (1.17–2.49) *			
Other CV events	1	1.71 (1.20–2.42) **	2.50 (1.79–3.49) **			
Death for all causes	1	1.00 (0.88–1.14)	0.94 (0.83–1.08)			
Combined MACE	1	1.49 (1.08–2.05) *	2.14 (1.58–2.90) **			
Model 1, HR (95% CI)						
Incident HF	1	1.23 (0.82–1.83)	1.39 (0.94–2.05)			
Other CV events	1	1.67 (1.18–2.37) **	2.17 (1.55–3.05) **			
Death for all causes	1	1.02 (0.37–2.83)	1.58 (0.62–4)			
Combined MACE	1	1.47 (1.07–2.03) *	1.86 (1.37–2.54) **			
Model 2, HR (95% CI)						
Incident HF	1	1.15 (0.77–1.72)	1.27 (0.86–1.88)		0.82	0.80
Other CV events	1	1.62 (1.14–2.30) *	1.99 (1.42–2.80) **		0.64	0.56
Death for all causes	1	0.98 (0.35–2.71)	1.42 (0.56–3.6)		0.89	0.82
Combined MACE	1	1.42 (1.03–1.96) *	1.72 (1.36–2.35) **		0.82	0.80

Model 1 = Clusters + diabetes, hypertension, and previous myocardial infarction.

Model 2 = Model 1 + LVEF.

The joint effect of LVEF and cluster assignment (interaction term) in the association with any outcome was not significant (*P* < 0.05).

CI, confidence interval; CV, cardiovascular; HF, heart failure; HR, hazard ratio; LVEF, left ventricular ejection fraction; MACE, major adverse CV events.

*P* < 0.05; ** *P* < 0.005.

Compared to Cluster 1, subjects in Cluster 3 had a significantly higher risk of MACE, particularly other CV events, in both unadjusted and adjusted models (*Table 4*). This group also showed a higher risk of developing HF [hazard ratio (HR): 1.70 (95% CI: 1.17–2.49), *P* < 0.05], but this association was fully attenuated in adjusted multivariate models.

Cluster 2 had an increased risk of other CV events and the combined endpoint of MACE compared to the reference cluster (Cluster 1), although to a lesser extent than Cluster 3. This association was independent of confounding factors.

The interaction between cluster label, LVEF, and any of the analysed clinical endpoints was not significant (*Table 4*), indicating that the differential risk across clusters was not influenced by changes in LVEF.

## Discussion

In the UK Biobank cohort, clustering analysis of clinical data identified three distinct categories of initially asymptomatic LVSD. Subjects in each cluster exhibited considerable variation in physical characteristics, lifestyle habits, CV risk factors, ECG parameters, and biomarker levels. Clusters also displayed different levels of CV impairments, linking clinical and imaging phenotypes. The identified phenogroups showed independent associations with CV outcomes, highlighting diverse risk profiles and clinical trajectories. These findings emphasize the significant heterogeneity amongst subjects developing LVSD and the importance of improved phenotyping, particularly in asymptomatic stages, for risk stratification and prognosis (*Graphical Abstract*).

Previous studies demonstrated clustering’s ability to identify clinically meaningful phenogroups in syndromes like chronic HF, offering insights into disease pathophysiology.^5,17^ Similarly, we demonstrated clustering’s feasibility to uncover unique patterns in available data, enhancing LVSD characterization and understanding. Identifying LVSD subgroups with similar progression patterns enables tailored treatment planning and improved prognosis.

### Distinct LVSD risk profiles

Three risk profiles of LVSD were identified, each associated with different lifestyle behaviours and CV risk factors. The risk of CV events increased progressively from Cluster 1 to Cluster 3, but there was no significant difference in all-cause death. Although co-morbid burden showed a gradual increase across the clusters, prevalent conditions such as myocardial infarction, stroke, and other organ diseases did not differ significantly. This suggests that multiple factors contribute to risk definition, with lifestyle behaviours playing a crucial role.

Cluster 1, the smallest subset, had the highest percentage of females with the healthiest diet habits. This group had the most favourable CV outcomes compared to the predominantly male clusters. This implies that non-modifiable factors, including genetic predisposition and sex-specific differences in CV risk factors and pathophysiological mechanisms, may influence risk definition.^18^ The low proportion of females in the cohort also confirms the higher prevalence of LVSD in men than in women in the general population.^3,19^

Subjects in Cluster 2, the intermediate-risk subset, had the highest level of physical activity, reflecting favourable effects on the CV system. They also had better blood pressure and heart rate control but higher consumption of processed food and alcohol compared to the lowest risk subset. These findings confirm the protective effect of exercise and highlight the benefits of combining healthy eating habits with physical activity for CV disease prevention.^20,18^ They also reinforce the strong link between processed food intake and CV risk, even in individuals with an otherwise healthy diet.^21^

Subjects in Cluster 3 had the highest burden of CV risk factors, unhealthiest lifestyle behaviours, worst cardiometabolic profile, and blood pressure control. This phenotype was associated with the highest risk of progression to HF (unadjusted model) and other CV events, confirming the role of modifiable risk factors as major drivers of CV diseases. These subjects had the highest adiposity and lean body mass indices, suggesting that non-adipose tissue significantly impacts CV risk, potentially affecting haemodynamics and inducing ventricular hypertrophy.^22^

The overall incidence of MACE was high in the whole cohort despite a mild reduction in LVEF [median LVEF: 47.6; interquartile range (IQR): 44.7–49.1]. These findings align with previous literature showing an increased risk of adverse outcomes in LVSD patients, even if with only mildly reduced LVEF, compared to individuals with normal LVEF.^23^ The MACE risk observed in this cohort is also significantly higher than what has been reported for UK Biobank participants with LVEF >55%, as indicated by a recent study.^24^ These findings highlight the importance of preventative actions to improve prognosis of LVSD, not guided merely by LVEF values but, as our study suggests, guided by the overall clinical risk profile.

### CV imaging patterns across the clusters

Distinct CV imaging patterns across the clusters reflected their different risk profiles (*Graphical Abstract*, Panel D). LV performance and cardiac mechanics deteriorated progressively, with increasing in LV adverse re-modelling from Cluster 1 to Cluster 3. Abnormal ventricular–arterial coupling patterns were also observed, indicating adaptive changes in LV structure and function in response to arterial load components.

Cluster 1, the lowest risk group, displayed minimal cardiac re-modelling, smallest bi-ventricular volumes, and better systolic function and myocardial deformation. Their vascular stiffening pattern featured reduced AoD and lower peripheral resistance. These findings might be attributed to the higher proportion of females in this group, known for more favourable re-modelling process and a higher predisposition towards diastolic dysfunction and HF with preserved LVEF.^25^

Cluster 2 had the lowest arterial load (highest TAC), intermediate systolic alterations. and LV re-modelling, with more eccentric LV geometry compared to those in Cluster 3. The compliance of small arteries, representing the resistive component of LV afterload, was reduced to a greater degree than the compliance of large vessels (AoD). This suggests a unique imaging phenotype of LVSD characterized by micro-vascular dysfunction, likely due to ageing and risk factors, and almost normal large vessel compliance, likely due to the positive effect of physical activity on haemodynamics.^26^ Such favourable effect on ventricular–arterial coupling might play a role in mitigating the risk of adverse CV events in this group.

Cluster 3 had the most adverse CV imaging phenotype, with the greatest LV re-modelling (highest M/V ratio indicating greater concentricity), poorest myocardial deformation and systolic function, and decreased arterial compliance. These alterations likely reflect the highest burden of CV risk factors^27^ and advanced ventricular–arterial decoupling, where myocardial functional impairment leads to a compensatory increase in SVR and arterial load, further predisposing to LV deterioration. This pattern resembles that observed in HF with reduced LVEF and is associated with poor CV outcomes.^9^ This phenotype had the worst prognosis, including a significantly increased risk of developing HF, driven by the highest burden of CV risk factors.

Our observations align with existing literature, linking adverse cardiac re-modelling with poor outcomes and HF progression. As observed in this study, an increase in cardiac re-modelling and vascular stiffening demonstrates progressive worsening of cardiac function and is associated with worse CV prognosis.^28^

### Clinical implications

We demonstrated that a comprehensive evaluation of readily available clinical data, which do not include CMR-derived measurements as they are not routinely available for all patients, can identify distinct risk profiles in LVSD, linked to specific imaging phenotypes. Our findings suggest that patients with high-risk clinical profiles, which correlate with adverse imaging phenotypes, should be prioritized for further in-depth imaging investigations. This strategy could potentially improve cost-efficiency and enhance the efficacy of preventive strategies.^29^ Risk stratification can facilitate tailored early interventions for different LVSD sub-types to improve prognostication. Lifestyle recommendations can reduce the risk of developing LVSD and adverse CV events for individuals resembling Clusters 1 and 2, whilst better management of co­-morbid conditions and lifestyle promotion may benefit those resembling Cluster 3.

### Strengths and limitations

To our knowledge, this is the first study applying clustering analysis of clinically available data to a large cohort of initially asymptomatic subjects to identify distinct LVSD phenotypes. Furthermore, we used CMR, which provides the most accurate and reproducible assessment of cardiac structure and function, to evaluate the CV changes associated with the discovered clinical sub-types of LVSD. However, several limitations of this analysis must be acknowledged.

First and foremost, the purpose of this study was not to propose a new classification of LVSD, as the obtained clusters may vary according to the characteristics of the study population and the available data. Instead, our results highlight the importance of a multi-dimensional evaluation of LVSD to guide personalized interventions based on the heterogeneity in key clinical characteristics within this condition.

Our study cohort was mainly composed of Caucasians with a relatively low burden of co-morbidities, likely reflecting the overall characteristics of the UK Biobank population and with a greater representation of males. Therefore, our results may not be generalizable to the entire population of LVSD patients.

Data on diet and other lifestyle habits, used as phenotypic features for clustering analysis, were assessed by self-reported questionnaires, therefore, may be subjected to bias which could lead to inaccuracy. We demonstrated the predictive utility of clustering using Cox regression analysis and successfully validated our findings by re-sampling. However, future studies that replicate our results in independent cohorts of LVSD might help demonstrate the generalizability of our data.

## Conclusions

Unbiased clustering analysis of baseline clinical data in asymptomatic patients revealed three distinct LVSD phenotypes differing in risk profile, CV imaging characteristics, and clinical trajectories. These findings underscore the heterogeneity within LVSD and the need for multi-dimensional characterization prior to symptom development, enabling personalized care programmes.

## Supplementary data

Supplementary data are available at *European Heart Journal - Cardiovascular Imaging* online.

## Supplementary Material

jead218_Supplementary_Data

## Data Availability

This research was conducted using the UK Biobank resource under access application 2964. UK Biobank will make the data available to all *bona fide* researchers for all types of health-related research that is in the public interest, without preferential or exclusive access for any persons. All researchers will be subject to the same application process and approval criteria as specified by UK Biobank. For more details on the access procedure, see the UK Biobank website: http://www.ukbiobank.ac.uk/register-apply/.
