# Supplementary material for: Phenotyping left ventricular systolic dysfunction in asymptomatic individuals for improved risk stratification
Source: Eur Heart J Cardiovasc Imaging. 2023 Sep 12;24(10):1363–73. doi: 10.1093/ehjci/jead218 (PMC10531121; doi:10.1093/ehjci/jead218)
Supplement: jead218_Supplementary_Data [file jead218_supplementary_data.docx]

**Supplemental Material**

1. **Supplemental Table 1.** Exclusion criteria for identifying the study population.
2. **Supplemental Table 2.** UK Biobank data sources used to identify the phenotypic features.
3. **Supplemental Table 3.** UK Biobank data sources used to identify clinical outcomes.
4. **Supplemental Table 4.** Baseline clinical and imaging characteristics of the study population.
5. **Supplemental Table 5.** Occurrence of adverse outcomes (incident events) in the whole study population.
6. **Supplemental Table 6.** Laboratory and electrocardiographic characteristics stratified by clusters.
7. **Supplemental Methods 1.** Phenotypic domains.
8. **Supplemental Methods 2.** CMR image acquisition and analysis.
9. **Supplemental Methods 3.** Statistical analysis.
10. **Supplemental References**

**Supplemental Table 1. Exclusion criteria for identifying the study population.**

| **Source** | **UK Biobank field** | **Description** |
| --- | --- | --- |
| ***Heart Failure*** |  |  |
| Self-reported condition | 20002 | heart failure/pulmonary oedema |
| ICD10 Summary diagnoses | 41270, 41280 | I50 Heart failure |
|  |  | I11.0 Hypertensive heart disease with (congestive) heart failure  I13.0 Hypertensive heart and renal disease with (congestive) heart failure  I13.2 Hypertensive heart and renal disease with both (congestive) heart failure and renal failure  K76.1 Chronic passive congestion of liver  J81 Pulmonary oedema |
| First occurrences | 131354 | Date first diagnosis: Heart failure |
|  | 131288 | Date first diagnosis: Hypertensive heart disease |
|  | 131292 | Date first diagnosis: Hypertensive heart and renal disease |
|  | 131670 | Date first diagnosis: Congestion of liver |
|  | 131524 | Date first diagnosis: Pulmonary oedema |
| ***Cardiomyopathies (any cause)*** | |  |
| Self-reported condition | 20002 | Cardiomyopathy |
| ICD10 Summary diagnoses | 41270, 41280 | I42 Cardiomyopathy |
|  |  | I43 Cardiomyopathy in diseases classified elsewhere |
|  |  | I25.5 Ischaemic cardiomyopathy |
| First occurrences | 131338 | Date first diagnosis: Cardiomyopathy |
|  | 131340 | Date first diagnosis: Cardiomyopathy in diseases classified elsewhere |
|  | 131306 | Date first diagnosis: Chronic ischaemic heart disease |
| ***Dyspnoea on exertion*** |  |  |
| Self-reported condition | 4717 | Shortness of breath walking on level ground |

**Supplemental Table 2. UK Biobank data sources used to identify the phenotypic features.**

| **Phenotypic domains** | **Phenotypic features** | **UK Biobank field** |
| --- | --- | --- |
| ***Socio-demographics*** |  |  |
|  | **Age (at baseline)** | 21003 |
|  | Ethnicity*^*^* | 21000 |
|  | **Sex^*^** | 31 |
|  | **Education level^*^** | 6138 |
|  | **Townsend deprivation index** | 189 |
| ***Physical measurements*** | |  |
|  | Body Mass Index (BMI) | 21001 |
|  | Body Surface Area (BSA) | 50,21002 |
|  | **Height** | 50 |
|  | Weight | 21002 |
|  | Waist circumference (WC) | 48 |
|  | Hip circumference (HC) | 49 |
|  | **Waist hip ratio (WHR)** | 48, 49 |
|  | **Waist height ratio (WHtR)** | 48, 50 |
|  | Fat mass (FM) | 23100 |
|  | Fat free mass (FFM) | 23101 |
|  | **Fat mass index (FMI)** | 23100, 50 |
|  | **Fat free mass index (FFMI)** | 23101, 50 |
|  | Basal metabolism rate (BMR) | 23105 |
|  | Body fat percentage (BF) | 23099 |
|  | **Systolic Blood Pressure (SBP)** | 4080 |
|  | **Diastolic Blood Pressure (DBP)** | 4079 |
|  | **Pulse rate (when reading automated blood pressure)** | 102 |
| ***Lifestyle factors*** | |  |
|  | **Smoking^*^** | 20116 |
|  | **Alcohol intake^*^** | 1558 |
|  | **Physical activity – IPAQ score** | 864, 874, 884, 894, 904, 914 |
|  | **Time watching TV, hours/day** | 1070 |
|  | **Time using computer, hours/day** | 1080 |
|  | **Sleep duration, hours/day** | 1160 |
|  | **Fruit intake, serving/day^*^** | 1309, 1319 |
|  | **Vegetable intake, serving/day^*^** | 1299, 1289 |
|  | **Oily fish intake, portion/week^*^** | 1329 |
|  | **Non oily fish intake, portion/week^*^** | 1339 |
|  | **Processed meat intake, portion/week^*^** | 1349 |
|  | **Red meat intake, portion/week^*^** | 1369,1379, 1389 |
|  | **Coffee intake, cups/day^*^** | 1498 |
|  | **Tea intake, cups/day^*^** | 1488 |
| ***Laboratory*** |  |  |
|  | **Calcium, mmol/L** | 30680 |
|  | **Creatinine, umol/L** | 30700 |
|  | **Urea, mmol/L** | 30670 |
|  | **Urate, umol/L** | 30880 |
|  | **eGFR, mL/min/1.73msq ^a^** | 21003, 31, 21000, 30700 |
|  | **Aspartate aminotransferase, U/L (AST)** | 30650 |
|  | **Alanine aminotransferase, U/L (ALT)** | 30620 |
|  | **Alkaline phosphatase, U/L (ALP)** | 30610 |
|  | **Gamma glutamyltransferase, U/L (GGT)** | 30730 |
|  | **Albumin, g/L** | 30600 |
|  | **Total bilirubin, umol/L** | 30840 |
|  | Red blood cell count, 10^12 cells/L (RBC) | 30010 |
|  | **White blood cell count, 10^9 cells/L (WBC)** | 30000 |
|  | **Platelet count, 10^9 cells/L** | 30080 |
|  | Mean corpuscular volume, femtolitres (MCV) | 30040 |
|  | **Mean corpuscular haemoglobin, picograms (MCH)** | 30050 |
|  | **Mean corpuscular haemoglobin concentration, gr/dL (MCHC)** | 30060 |
|  | Haematocrit percentage, % (HT) | 30030 |
|  | **Haemoglobin concentration, gr/dL** | 30020 |
|  | **Red blood cell distribution width, % (RDW)** | 30070 |
|  | Cholesterol, mmol/L | 30690 |
|  | **HDL cholesterol, mmol/L** | 30760 |
|  | **LDL cholesterol, mmol/L** | 30780 |
|  | **Triglycerides, mmol/L** | 30870 |
|  | Microalbumin in urine, mg/L | 30500 |
|  | **Sodium in urine, mmol/L** | 30530 |
|  | **Potassium in urine, mmol/L** | 30520 |
|  | **Creatinine in urine, micromole/L** | 30510 |
|  | **C-reactive protein mg/L (CRP)** | 30710 |
|  | **Vitamin D, nmol/L** | 30890 |
|  | **Glycated haemoglobin (HbA1c), mmol/mol** | 30750 |
| ***Electrocardiography*** |  |  |
|  | **P duration, ms** | 12338 |
|  | **QRS duration, ms** | 12340 |
|  | PQ interval, ms | 22330 |
|  | **QT interval, ms** | 22331 |
|  | **QTc interval, ms** | 22332 |
|  | RR interval, ms | 22333 |
|  | **PP interval, ms** | 22334 |
|  | P axis, degrees | 22335 |
|  | **R axis, degrees** | 22336 |
|  | **T axis, degrees** | 22337 |
|  | **Ventricular rate, bpm** | 12336 |

Bolded phenotypic features are those used for clustering analyses after filtering for missingness, and collinearity.

^*^ Categorical variables. ^a^ Glomerular Filtration Rate (GRF) was estimated using the EPI creatinine equation.

**Supplemental Table 3. UK Biobank data sources used to identify clinical outcomes.**

| **Source** | | **UK Biobank field** | | **Description** |
| --- | --- | --- | --- | --- |
| ***Heart Failure (all causes)*** | |  | |  |
| Self-reported condition | | 20002 | | Cardiomyopathy |
|  | |  | | heart failure/pulmonary oedema |
| ICD10 Summary diagnoses | | 41270, 41280 | | I50 Heart failure  I42 Cardiomyopathy  I43 Cardiomyopathy in diseases classified elsewhere |
|  | |  | | I11.0 Hypertensive heart disease with (congestive) heart failure  I13.0 Hypertensive heart and renal disease with (congestive) heart failure  I13.2 Hypertensive heart and renal disease with both (congestive) heart failure and renal failure  I25.5 Ischaemic cardiomyopathy  K76.1 Chronic passive congestion of liver  J81 Pulmonary oedema |
| First occurrences | | 131354 | | Date first diagnosis: Heart failure |
|  | | 131338 | | Date first diagnosis: Cardiomyopathy |
|  | | 131340 | | Date first diagnosis: Cardiomyopathy in diseases classified elsewhere |
|  | | 131288 | | Date first diagnosis: Hypertensive heart disease |
|  | | 131292 | | Date first diagnosis: Hypertensive heart and renal disease |
|  | | 131306 | | Date first diagnosis: Ischaemic cardiomyopathy |
|  | | 131670 | | Date first diagnosis: Congestion of liver |
|  | | 131524 | | Date first diagnosis: Pulmonary oedema |
| ***Myocardial infarction*** |  | |  | |
| Self-reported illness | | 20002 | | heart attack/myocardial infarction |
| ICD10 Summary diagnoses | | 41270, 41280 | | I21 Acute myocardial infarction |
|  | |  | | I22 Subsequent myocardial infarction |
|  | |  | | I23 Certain current complications following acute myocardial infarction |
|  | |  | | I24.1 Dressler's syndrome |
|  | |  | | I25.2 Old myocardial infarction |
| First occurrences | | 131298 | | Date first diagnosis: Acute myocardial infarction |
|  | | 131300 | | Date first diagnosis: Subsequent myocardial infarction |
|  | | 131302 | | Date first diagnosis: Certain current complications following acute myocardial infarction |
|  | | 131304 | | Date first diagnosis: Other acute ischaemic heart diseases |
|  | | 131306 | | Date first diagnosis: Chronic ischaemic heart disease |
| Diagnosed by doctor | | 3894 | | Age heart attack diagnosed |
| Algorithmically defined | | 42000 | | Date of myocardial infarction |
| ***Stroke*** | |  | |  |
| Self-reported illness | | 20002 | | stroke |
| ICD10 Summary diagnoses | | 41270, 41280 | | I60 Subarachnoid haemorrhage |
|  | |  | | I61 Intracerebral haemorrhage |
|  | |  | | I62 Other nontraumatic intracranial haemorrhage |
|  | |  | | I63 Cerebral infarction |
|  | |  | | I64 Stroke, not specified as haemorrhage or infarction |
| First occurrences | | 131360 | | Date first diagnosis: Subarachnoid haemorrhage |
|  | | 131362 | | Date first diagnosis: Intracerebral haemorrhage |
|  | | 131364 | | Date first diagnosis: Other nontraumatic intracranial haemorrhage |
|  | | 131366 | | Date first diagnosis: Cerebral infarction |
|  | | 131368 | | Date first diagnosis: Stroke, not specified as haemorrhage or infarction |
| Diagnosed by doctor | | 4056 | | Age stroke diagnosed |
| Algorithmically defined | | 42006 | | Date of stroke |
| ***Peripheral vascular diseases*** | |  | |  |
| Self-reported illness | | 20002 | | peripheral vascular disease  aortic aneurysm |
| ICD10 Summary diagnoses | | 41270, 41280 | | I70 Atherosclerosis |
|  | |  | | I71 Aortic aneurysm and dissection |
|  | |  | | I72 Other aneurysm |
|  | |  | | I73.9 Peripheral vascular disease, unspecified |
| First occurrences | | 131382 | | Date first diagnosis: Aortic aneurysm and dissection |
|  | | 131384 | | Date first diagnosis: Other aneurysm |
|  | | 131386 | | Date first diagnosis: Other peripheral vascular diseases |
| ***Cardiac arrhythmia*** | |  | |  |
| Self-reported illness | | 20002 | | svt / supraventricular tachycardia |
|  | |  | | atrial fibrillation |
|  | |  | | atrial flutter |
| ICD10 Summary diagnoses | | 41270, 41280 | | I44.1 Atrioventricular block, second degree |
|  | |  | | I44.2 Atrioventricular block, complete |
|  | |  | | I46.0 Cardiac arrest with successful resuscitation |
|  | |  | | I46.1 Sudden cardiac death, so described |
|  | |  | | I46.9 Cardiac arrest, unspecified |
|  | |  | | I47.0 Re-entry ventricular arrhythmia |
|  | |  | | I47.1 Supraventricular tachycardia |
|  | |  | | I47.2 Ventricular tachycardia |
|  | |  | | I47.9 Paroxysmal tachycardia, unspecified |
|  | |  | | I48.0 Paroxysmal atrial fibrillation |
|  | |  | | I48.1 Persistent atrial fibrillation |
|  | |  | | I48.2 Chronic atrial fibrillation |
|  | |  | | I48.3 Typical atrial flutter |
|  | |  | | I48.4 Atypical atrial flutter |
|  | |  | | I48.9 Atrial fibrillation and atrial flutter, unspecified |
|  | |  | | I49.0 Ventricular fibrillation and flutter |
|  | |  | | I49.2 Junctional premature depolarisation |
|  | |  | | I49.3 Ventricular premature depolarisation |
|  | |  | | I49.4 Other and unspecified premature depolarisation |
| First occurrences | | 131346 | | Date first diagnosis: Cardiac arrest |
|  | | 131348 | | Date first diagnosis: Paroxysmal tachycardia |
|  | | 131350 | | Date first diagnosis: Atrial fibrillation and flutter |
| ***Death (any cause)*** | |  | |  |
| Death register | | 40000 | | Date of death |
| ***Cardiovascular death*** | |  | |  |
| Underlying (primary) cause of death: ICD-10 | | 40001 | | Diseases of the circulatory system: I05-I89 |

**Supplemental Table 4. Baseline clinical and imaging characteristics of the study population.**

| **Baseline characteristics** | **All cohort**  **(n = 1,563)** |
| --- | --- |
| ***Demographics*** | |
| Age, years | 56.25±7.56 |
| Female, n (%) | 364(23.29) |
| Ethnicity, n (%) |  |
| *White* | 1518(97.4) |
| *Mixed* | 3(0.19) |
| *Asian* | 12(0.77) |
| *Black* | 17(1.09) |
| *Others* | 9(0.55) |
| ***Comorbidities, n (%)*** | |
| Smoking history | 699(44.72) |
| Previous myocardial infarction | 40(2.56) |
| Stroke | 20(1.28) |
| Chronic obstructive pulmonary disease | 16(1.0) |
| Asthma | 219(14.01) |
| Atrial fibrillation | 49(3.13) |
| Peripheral artery disease | 13(0.83) |
| Hypertension | 460(29.4) |
| Diabetes | 83(5.31) |
| Hypercholesterolemia | 312(20.1) |
| CKD | 14(0.9) |
| ***Physical measurements*** | |
| Body mass index, Kg/m^2^ | 26.71(24.46-29.30) |
| Waist hip ratio | 0.90 (0.85-0.95) |
| Systolic blood pressure, mmHg | 138 ±16.3 |
| Diastolic blood pressure, mmHg | 83.8±9.9 |
| ***Laboratories*** | |
| eGFR, mL/min/1.73m² | 91.41 ±11.76 |
| Haemoglobin, g/dl | 14.58 ±1.15 |
| LDL cholesterol, mmol/L | 3.53 ±0.79 |
| Triglycerides, mmol/L | 1.58 (1.12-2.12) |
| ***Resting ECG*** | |
| QRS duration, ms | 96 (86-100) |
| QTc interval, ms | 424 ±25 |
| ***CMR indices of cardiac structure and function*** | |
| LVEDVi (ml/m^2^) | 86.87±18.47 |
| LVESVi (ml/m^2^) | 45.12(39.94-51.96) |
| LVSVi (ml/m^2^) | 39.81±8.36 |
| LVMi (g/m^2^) | 50.92(44.80-57.22) |
| LVEF (%) | 47.57(44.75-49.06) |
| RVEDVi (ml/m^2^) | 86±16.76 |
| RVESVi (ml/m^2^) | 43.87±10.55 |
| RVSVi (ml/m^2^) | 42.13±9.70 |
| RVEF (%) | 49.03±7.04 |
| M/V ratio (g/ml) | 0.59(0.53-0.65) |
| LVGFI (%) | 35.24(32.59-37.44) |
| ***CMR-derived indices of arterial function*** | |
| AoD (x10^-3^/mmHg) | 1.12(0.67-1.87) |
| Indexed SVR (mmHg.min.L^−1^.m^2^) | 29.62 (23.67 - 36.35) |
| Indexed TAC (mL/mmHg/m^2^) | 0.66(0.52 - 0.84) |
| ***CMR-derived indices of cardiac mechanics*** | |
| GLS (%) | -14.77(16.24 - 12.89) |
| GCS (%) | -14.51(15.69 - 12.92) |
| GRS (%) | 20.62±4.86 |
| Torsion (degrees) | 0.71±0.48 |

Values are mean (±standard deviation) when continuous or number (percentage) when categorical. Data are presented as median (interquartile range) where absolute skew is ≥ 0.9. *AoD = aortic distensibility, GCS = global circumferential strain, GLS = global longitudinal strain, GRS = global radial strain, LVEDVI = left ventricular end-diastolic volume index, LVEF = left ventricular ejection fraction, LVMI = left ventricular mass index, LVESVI = left ventricular end-systolic volume index, LVGFI = left ventricle global function index, LVSV= left ventricular stroke volume index, M/V = LV mass-to-volume ratio, RVEDVI = right ventricular end-diastolic volume index, RVEF = right ventricular ejection fraction, RVESVI = right ventricular end-systolic volume index, RVSVI = right ventricular stroke volume index, SVR = systemic vascular resistance, TAC= total arterial compliance.*

**Supplemental Table 5. Occurrence of adverse outcomes (incident events) in the whole study population.**

| **Outcomes, n (%)** | **All cohort**  **(n = 1,563)** |
| --- | --- |
| HF | 215(13.9) |
| Other CV events | 336(21.5) |
| All-cause death | 36(2.30) |
| Combined MACE | 364(23.2) |

*HF = heart failure, CV = cardiovascular, MACE = major adverse cardiovascular events.*

**Supplemental Table 6. Laboratory and electrocardiographic characteristics stratified. by clusters**

|  | **Cluster 1**  **(n=348)** | **Cluster 2**  **(n=596)** | **Cluster 3**  **(n=619)** | **P Value** | **Cluster 1 vs**  **Cluster 2** | **Cluster 1 vs**  **Cluster 3** | **Cluster 2 vs**  **Cluster 3** |
| --- | --- | --- | --- | --- | --- | --- | --- |
| ***Laboratories*** | | | | |  |  |  |
| Calcium, mmol/L | 2.4±0.1 | 2.36±0.09 | 2.38±0.08 | **<0.001** | 0.97 | 0.001 | **<0.001** |
| Creatinine, umol/L | 64.5±9.7 | 80.99±11.61 | 80.58±12.64 | **<0.001** | **<0.001** | **<0.001** | 0.56 |
| Urea, mmol/L | 5.1(4.3-5.7) | 5.5(4.8-6.2) | 5.5(4.8-6.3) | **<0.001** | **<0.001** | **<0.001** | 0.86 |
| Urate, umol/L | 263±53.3 | 335.79±57.99 | 371.7±67.74 | **<0.001** | **<0.001** | **<0.001** | **<0.001** |
| eGFR, mL/min/1.73m² | 93.4±11.3 | 91.3±11.36 | 90.43±12.25 | **<0.001** | **0.007** | **<0.001** | 0.20 |
| Aspartate aminotransferase, U/L | 22.4(19.7-26.3) | 26(22.8-28.8) | 27.3(23.8-32.2) | **<0.001** | **<0.001** | **<0.001** | **0.01** |
| Alanine aminotransferase, U/L | 16.7(13.4-21.2) | 21.4(16.8-25.3) | 28(22.9-38.2) | **<0.001** | **<0.001** | **<0.001** | **<0.001** |
| Alkaline phosphatase, U/L | 80.2(67.1-90.4) | 76.9(66-84.5) | 80.8(70.2-94.3) | **<0.001** | **0.002** | **0.03** | **<0.001** |
| Gamma glutamyl transferase, U/L | 20.1(15.9-28.2) | 26.2(20.4-37.3) | 39(30.2-58.1) | **<0.001** | **<0.001** | **<0.001** | **<0.001** |
| Albumin, g/L | 44.8±2.4 | 45.3±2.28 | 45.76±2.47 | **<0.001** | **<0.001** | **<0.001** | **<0.001** |
| Total bilirubin, umol/L | 7.5(5.7-10.1) | 10.1(8.2-11.9) | 9.3(7.5-11.3) | **<0.001** | **<0.001** | **<0.001** | **0.001** |
| White blood cell count, 10^9 cells/L | 6.5(5.5-7.4) | 5.9(4.9-6.8) | 6.7(5.9-7.9) | **<0.001** | **<0.001** | **0.001** | **<0.001** |
| Platelet count, 10^9 cells/L | 259(230-304) | 226(196-254) | 238(204-265) | **<0.001** | **<0.001** | **<0.001** | **<0.001** |
| Mean corpuscular haemoglobin, pg | 31.2±1.8 | 31.74±1.6 | 31.65±1.49 | **<0.001** | **<0.001** | **<0.001** | 0.32 |
| Mean corpuscular haemoglobin concentration, g/dl | 34.3(33.8-34.9) | 34.6(33.9-35.2) | 34.6(34.2-35.3) | **<0.001** | **<0.001** | **<0.001** | 0.5 |
| Haemoglobin, g/dl | 13.4±0.9 | 14.71±0.92 | 15.11±0.99 | **<0.001** | **<0.001** | **<0.001** | **<0.001** |
| Red blood cell distribution width, % | 13.4(12.9-13.9) | 13.3(12.9-13.7) | 13.3(12.9-13.7) | 0.09 | **0.03** | 0.12 | 0.45 |
| HDL cholesterol, mmol/L | 1.5(1.3-1.8) | 1.3(1.2-1.5) | 1.2(1-1.4) | **<0.001** | **<0.001** | **<0.001** | **<0.001** |
| LDL cholesterol, mmol/L | 3.5±0.8 | 3.44±0.74 | 3.62±0.82 | **<0.001** | 0.08 | 0.06 | **<0.001** |
| Triglycerides, mmol/L | 1.3(1-1.8) | 1.4(1-1.8) | 1.9(1.5-2.8) | **<0.001** | **0.04** | **<0.001** | **<0.001** |
| Sodium in urine, mmol/L | 59.6±34.8 | 74.79±40.96 | 94.16±42.54 | **<0.001** | **<0.001** | **<0.001** | **<0.001** |
| Potassium in urine, mmol/L | 56.9±33.8 | 68.17±33.44 | 72.79±33.35 | **<0.001** | **<0.001** | **<0.001** | **0.02** |
| Creatinine in urine, mmol/L | 5531(3452-9091) | 8579(5638-12840) | 10489(7629-14710) | **<0.001** | **<0.001** | **<0.001** | **<0.001** |
| C-reactive protein, mg/L | 1.2(0.6-2.2) | 0.9(0.5-1.7) | 1.7(1-2.8) | **<0.001** | **<0.001** | **<0.001** | **<0.001** |
| Vitamin D, nmol/L | 50.7±20.8 | 54.49±18.76 | 46.12±19.28 | **<0.001** | **0.003** | **<0.001** | **<0.001** |
| Glycated haemoglobin, mmol/mol | 35(32.4-37.1) | 34.4(32-36.1) | 35.7(33.5-38.6) | **<0.001** | **0.007** | **<0.001** | **<0.001** |
| ***Resting ECG*** | | | | |  |  |  |
| P duration, ms | 95.7±14.5 | 99.22±15.45 | 98.97±15.39 | **0.001** | **<0.001** | **0.001** | 0.77 |
| QRS duration, ms | 88(80-97) | 97.2(90-102) | 96(86-100) | **<0.001** | **<0.001** | **<0.001** | **<0.001** |
| QT interval, ms | 416.4±28.9 | 424.52±27.99 | 405.67±31.97 | **<0.001** | **<0.001** | **<0.001** | **<0.001** |
| QTc interval, ms | 430.4±23.2 | 417.23±23.98 | 427.5±26.36 | **<0.001** | **<0.001** | 0.08 | **<0.001** |
| PP interval, ms | 928.3±166.7 | 1034.14±162.2 | 889.54±178.4 | **<0.001** | **<0.001** | **<0.001** | **<0.001** |
| R axis, degrees | 22.6±32.8 | 19.27±32.13 | 12.67±31.88 | **<0.001** | 0.12 | **<0.001** | **<0.001** |
| T axis, degrees | 38(31.7-50.2) | 38(27.7-44) | 38(20-39) | **<0.001** | **0.02** | **0.005** | **0.01** |
| Ventricular rate, bpm | 64.4(58.7-71) | 58(53-64.5) | 65(63-75) | **<0.001** | **<0.001** | **<0.001** | **<0.001** |

Values are presented as mean (± standard deviation) and as median (interquartile range) where absolute skew is ≥ 0.9. The P Value indicates comparisons of variables across clusters and bold values indicate statistical significance (p <0.05).

**Supplemental Methods 1. Phenotypic domains**

The phenotypic domains used for clustering included information on socio-demographics, physical measurements, health-related behaviours, electrocardiographic parameters, and laboratory data. A complete list of features per each phenotypic domain and their corresponding UK Biobank research codes are given in **Supplemental Table 2**. In addition, details on how the variables were assessed can be found in the UK Biobank Data Showcase (<https://www.ukbiobank.ac.uk/data-showcase/>).

Briefly, socio-demographics included information on age, sex, ethnicity, Townsend deprivation index (a socio-economic measure based on the area of residence) and education level, categorized as high/intermediate/low, as described in a previous publication (1).

Physical measurements were also collected, including anthropometrics, blood pressure, and pulse rate. Specifically, we considered manual body measures (e.g., waist and hip circumference, height) and body composition measures assessed by bioimpedance using the Tanita BC-418 MA analyzer according to a defined protocol (<https://biobank.ndph.ox.ac.uk/showcase/ukb/docs/body_composition.pdf>). Bioimpedance was used to estimate fat mass (FM) and fat-free mass (FFM) and thereafter calculate FM index (FMI; FM/height^2^) and FFM index (FFMI; FFM/height^2^) as described in a previous publication (2). Waist-hip ratio (WHR; waist circumference/hip circumference) and waist-height ratio (WHtR; waist circumference /height^2^) were also calculated as additional indices of body fat distribution (3). Blood pressure measurements and pulse rate were obtained using Omron Digital blood pressure monitor according to a publicly accessible protocol (<https://biobank.ndph.ox.ac.uk/ukb/ukb/docs/Bloodpressure.pdf>).

Participants' health-related behaviours were estimated based on their diet quality, physical activity level, sedentary time (time spent on tv and computer), sleep duration and other lifestyle behaviours (including smoking, tea, coffee, and alcohol intake) using selected UK Biobank research codes (**Supplemental Table 2**).

Diet quality was estimated from the intake frequency of a range of food items over the preceding year reported by participants through a touchscreen dietary questionnaire at the baseline visit. Fruits (fresh and dried) and vegetables (raw or cooked) intakes were expressed as servings per day (one piece of fruit and two heaped tablespoons of vegetables, respectively, were considered as one serving), whilst the consumption of unprocessed red meat (beef, pork, lamb/mutton), processed red meat (such as bacon, ham, sausages, meat pies, kebabs, burgers, chicken nuggets), oily fish and non-oily fish were each converted into weekly intake equivalents and then categorized, as reported in a previous publication (4).

Physical activity level was expressed in metabolic equivalent (MET) minutes/week. It was estimated by weighting different forms of activity (walking, moderate, or vigorous) by their energy requirements based on values derived from the International Physical Activity Questionnaire (IPAQ) study (5).

Sedentary time, sleep duration, tea and coffee intake (both expressed as cups per day), alcohol intake frequency (never, special occasions only, 1–3 times per month, 1–2 times per week, 3–4 times per week, and daily or almost daily), and smoking status (never smoker vs previous or current smoker) were based on self-report.

Twelve-lead (at-rest) electrocardiogram (ECG) measurement was performed at an Imaging assessment centre for UK Biobank using the ECG GE Cardiosoft program and according to a defined protocol (<https://biobank.ndph.ox.ac.uk/showcase/ukb/docs/12lead_ecg_explan_doc.pdf>).

Laboratory data included a range of key biomarkers collected at baseline visits in all participants with an available biological sample and according to a defined protocol (<https://biobank.ndph.ox.ac.uk/showcase/ukb/docs/biomarker_issues.pdf>). These variables were selected because they represent established diagnostic measures and risk factors for cardiovascular diseases.

**Supplemental Methods 2. CMR image acquisition and analysis**

The acquisition protocol of CMR imaging in UK Biobank has been described in detail by Petersen et al. (6). In brief, the CMR images were acquired with a 1.5 Tesla scanner (MAGNETOM Aera, Syngo Platform VD13A, Siemens Healthcare, Erlangen, Germany) with 48 receiver channels, an 18 channels anterior body surface coil used in combination with 12 elements of an integrated spine coil enabling 32 channel imaging and ECG gating for cardiac synchronization. Scans were performed in dedicated UK Biobank imaging centers using uniform staff training and equipment, according to the above protocol. The cardiac assessment included a combination of long axis (LAX) cines (horizontal long-axis – HLA, vertical long-axis – VLA, and left ventricular outflow tract –LVOT cines, both sagittal and coronal) and a complete short-axis (SAX) stack covering both left and right ventricles (LV, RV) acquired using balanced steady-state free precession sequences. All cine images were acquired at one slice per breath-hold.

CMR image segmentation was performed manually in the initial ~5,000 UK Biobank studies by two image-analysis core laboratories using CVI42 version 5.1.1 (Circle Cardiovascular Imaging Inc, Calgary, Canada) as previously described (7). This expert-annotated dataset was then used to develop an automated image analysis pipeline with inbuilt quality control, validated in a large sample of 32,000 UK Biobank CMR studies (8).

Conventional CMR indices of cardiac function and structure (LV and RV volumes in end-diastole, end-systole and stroke volumes; LV mass; LV and RV ejection fraction) were extracted using the automated image analysis pipeline described above (8). Additional cardiac indices able to capture changes in myocardial function in relation to structural chamber remodeling were analyzed, including LV mass-to-volume (M/V) ratio and LV global function index (LVGFI). M/V ratio was calculated as LV mass divided by LV end-diastolic volume. Increased M/V ratio has been previously linked with cardiac remodeling and poorer cardiovascular outcomes (9). LVGFI is a validated measure of LV cardiac performance that integrates structural components of adverse myocardial remodeling into LV function assessment, for which the formula is described elsewhere (10). Higher LVGFI indicates better LV cardiac performance and provides incremental value over LV ejection fraction in predicting adverse cardiovascular events (11).

Aortic distensibility, total arterial compliance (TAC), and systemic vascular resistance (SVR) derived from CMR were considered descriptors of arterial health. AoD, is a measure of local aortic compliance, and is considered the major contributor to arterial stiffness (12,13). TAC represents a measure of global arterial stiffness reflecting the effect of both large and small arteries’ properties; SVR, instead, represent the resistive component of the LV afterload, largely determined by the small vessel tone and depending on microvascular properties.

AoD was estimated by measuring the relative change in the area of the thoracic aorta on CMR images divided by central pulse pressure using Vicorder^®^ readings at the time of scanning (14). Aortic distensibility values were obtained from a previous analysis of a large subset of the UK Biobank imaging studies using a fully automated image analysis pipeline embedded with purpose-designed quality control (15). CMR values of cardiac output and stroke volume were used to estimate TAC (LV stroke volume/central pulse pressure) and SVR (mean arterial pulse pressure/cardiac output) (16). As the arterial load is highly dependent on body size, we indexed TAC and SVR for body surface area (BSA) as reported in a previous publication (17,18).

Left ventricle strain (tissue tracking) was analyzed using an automated tool (CVI42 version 5.13.7, Circle Cardiovascular Imaging Inc, Calgary, Canada). Long-axis cine images were tracked to derive global longitudinal strain (GCS), whilst SAX cine images were used to derive

global circumferential strain (GCS), global radial strain (GRS), and torsion of the LV. Images were first visually inspected to retain only those deemed of good quality. The number of participants with the strain values available after quality control were thus as follow: for GLS, n =1,523; for GCS, GRS, and torsion, n = 1,334.

**Supplemental Methods 3. Statistical analysis**

The main objective of this study was to identify clusters of left ventricular systolic dysfunction (LVSD) patients based on phenotypic data. The method that we used is outlined as follows.

*Selecting the phenotypic variables for clustering*

Seventy-eight baseline variables representing key characteristics of the study population (phenotypic domains), including socio-demographics, health-related lifestyle habits, physical characteristics, electrocardiographic parameters, and laboratory data, were initially scrutinized. The phenotypic domains included both continuous (numeric) and categorical features.

Next, variables with >30% missingness were excluded from our analysis (namely PQ duration, P axis, and microalbumin in urine), whilst the remainder were handled using mean (numeric variables) or most common value (categorical variables) imputation. Ethnicity was also not used for clustering as the study cohort mainly comprised Caucasians (97%). Furthermore, the obtained clusters did not differ significantly in ethnicity distribution, confirming that this category was irrelevant for clustering.

Subsequently, highly correlated variables potentially increasing the algorithm's complexity, thus increasing the risk of errors, were filtered, keeping only the most informative with the least missingness for clustering. While the categorical variables did not contain redundant information, the correlation matrix showed high correlations across large numbers of continuous features (**Figure S1** below). Therefore, the continuous variables that were correlated at *r* > 0.8 were filtered, leaving only the minimally redundant ones. Sixty candidate mixed features (48 continuous and 12 categorical) covering all the phenotypic domains were thus finally selected and used for subsequent clustering (**Supplemental Table 2** shows all phenotypic domains with the sixty features finally selected for clustering analysis in bold). The whole process of selecting the phenotypic variables for clustering is summarized in **Figure S2** below.


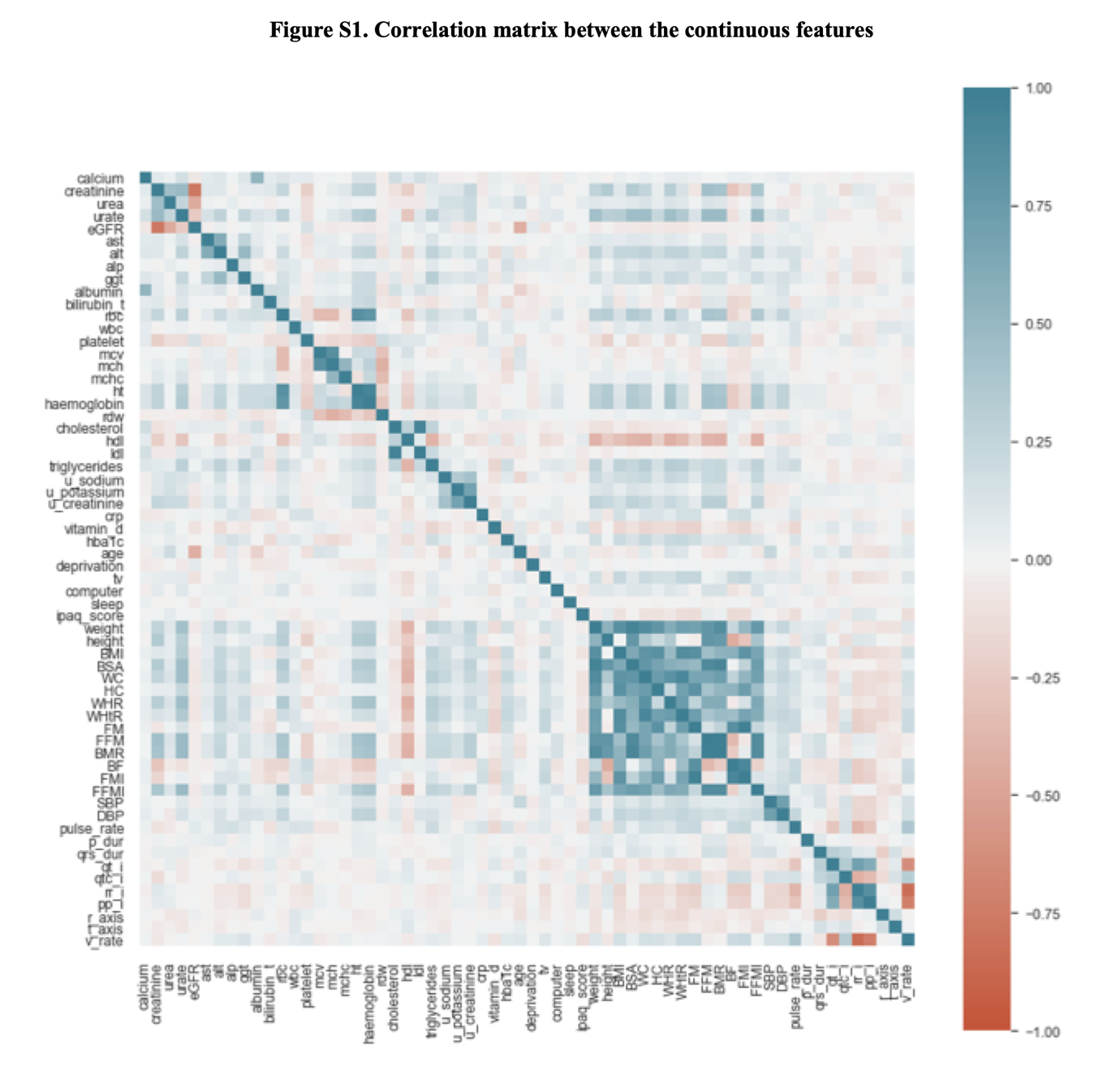


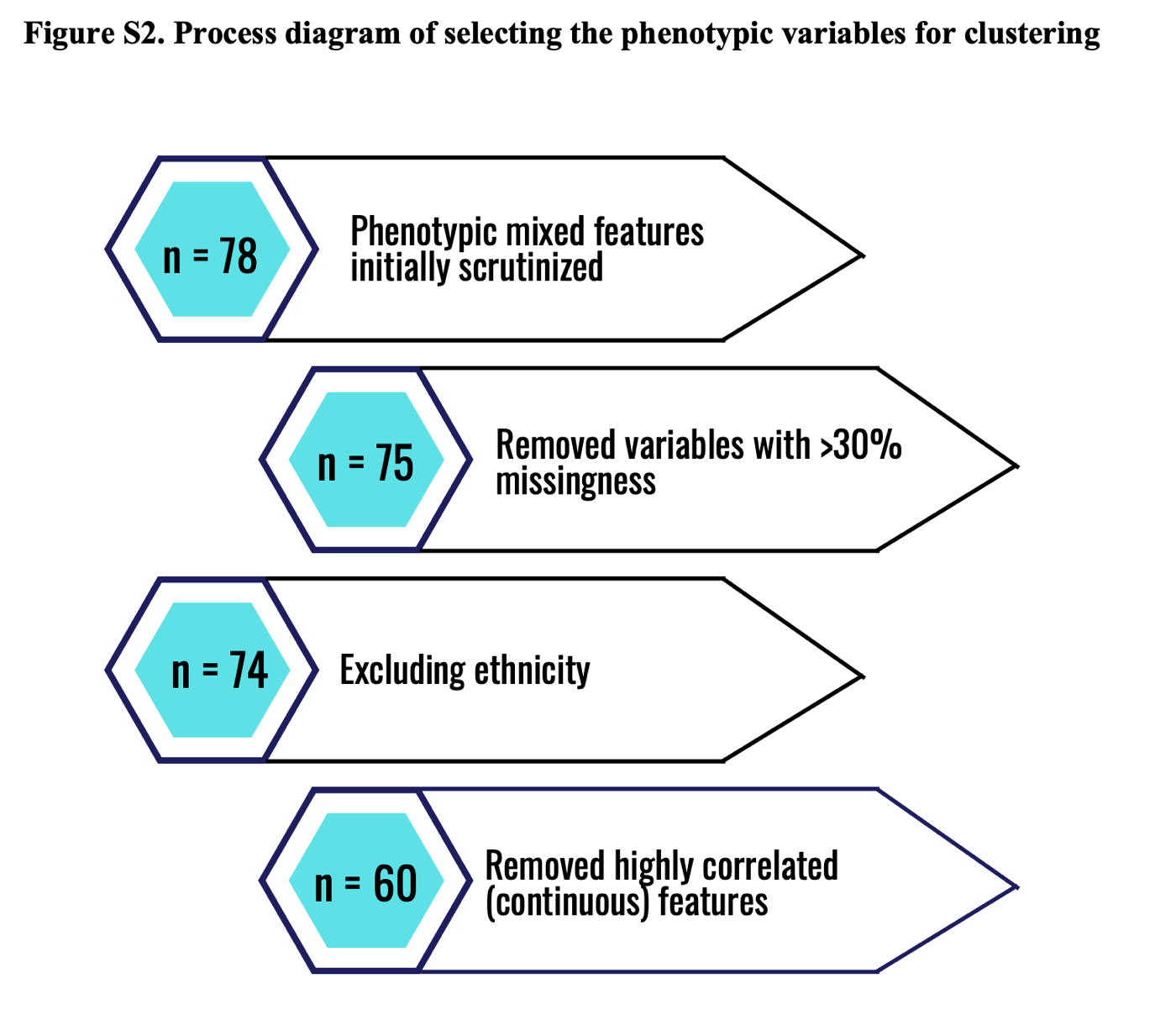


*Clustering analysis*

Clustering is an unsupervised machine learning technique that aims to identify hidden patterns within data by portioning unlabeled data into groups based on similar characteristics. It can thus be used to characterize disease phenotypes without the need for a priori assumptions about classification.

As our phenotypic domains contained mixed data, we selected k-prototype clustering (19) because of its efficiency in dealing with large datasets with mixed variable types (20). The k-prototypes algorithm, first published by Huang (19), integrates both the k-means (for numeric data) and the k-modes (for categorical data) algorithms to handle mixed data. The distance metric used to assign the observations to the clusters combine the Euclidean distance for numeric attributes with the simple matching distance for categorical attributes.

For this analysis, we used the *kprototypes* function from the *kmodes* package (0.12.2) implemented in Python. The continuous features were normalized to zero mean and unit variance to account for different measurement scales before fitting them to the model. The categorical variables instead did not require any encoding as the k-prototypes algorithm could handle them itself.

The cost function, which is the sum of all the dissimilarities between the clusters, was used to determine the optimal number of clusters through the Elbow Method. Specifically, we calculated the cost for each value of clusters (k) in a given range (1 - 10). We then identified the optimal number of clusters (k) that balances minimizing the variance within clusters as the point where an elbow-like bend with a lesser cost was observed. The identification of the elbow point from the plotted curve was further confirmed using the *KneeLocator* function from the *kneed* package (0.8.1) implemented in Python (21). The Elbow Method by plotting the cost as a function of the number of clusters indicated that a three-cluster model best fit the dataset (**Figure S3** below**)**. Clustering of the 60 phenotypic features was then repeated using the optimal k-values previously identified (k =3), and using the ‘Huang’ approach to initialize the clusters (19). We further examined the individual features across the clusters to ensure that the selected k-value produced clinically meaningful clusters with distinct characteristics in the main phenotypic features. All clustering was performed blinded to clinical outcomes and CMR metrics, as the latter two served for their clinical validation.

A resampling-based method was used to assess the stability of the clustering results with respect to sampling variability (22). We randomly resampled 80% of the entire cohort, and repeated data-processing and clustering for a total of 5 resamplings. The resulting phenogroups from each resampling were compared to the original phenogroups from the entire cohort (e.g., clusters 1, 2 and 3 from each resampling were compared to the original clusters 1, 2 and 3 from the whole cohort, respectively). Nearly all characteristics, including clinical, CMR-derived metrics, and outcomes, were similar between the examined phenogroups. This suggests that our grouping was not due to random allocation but instead captured existing patterns within the dataset.


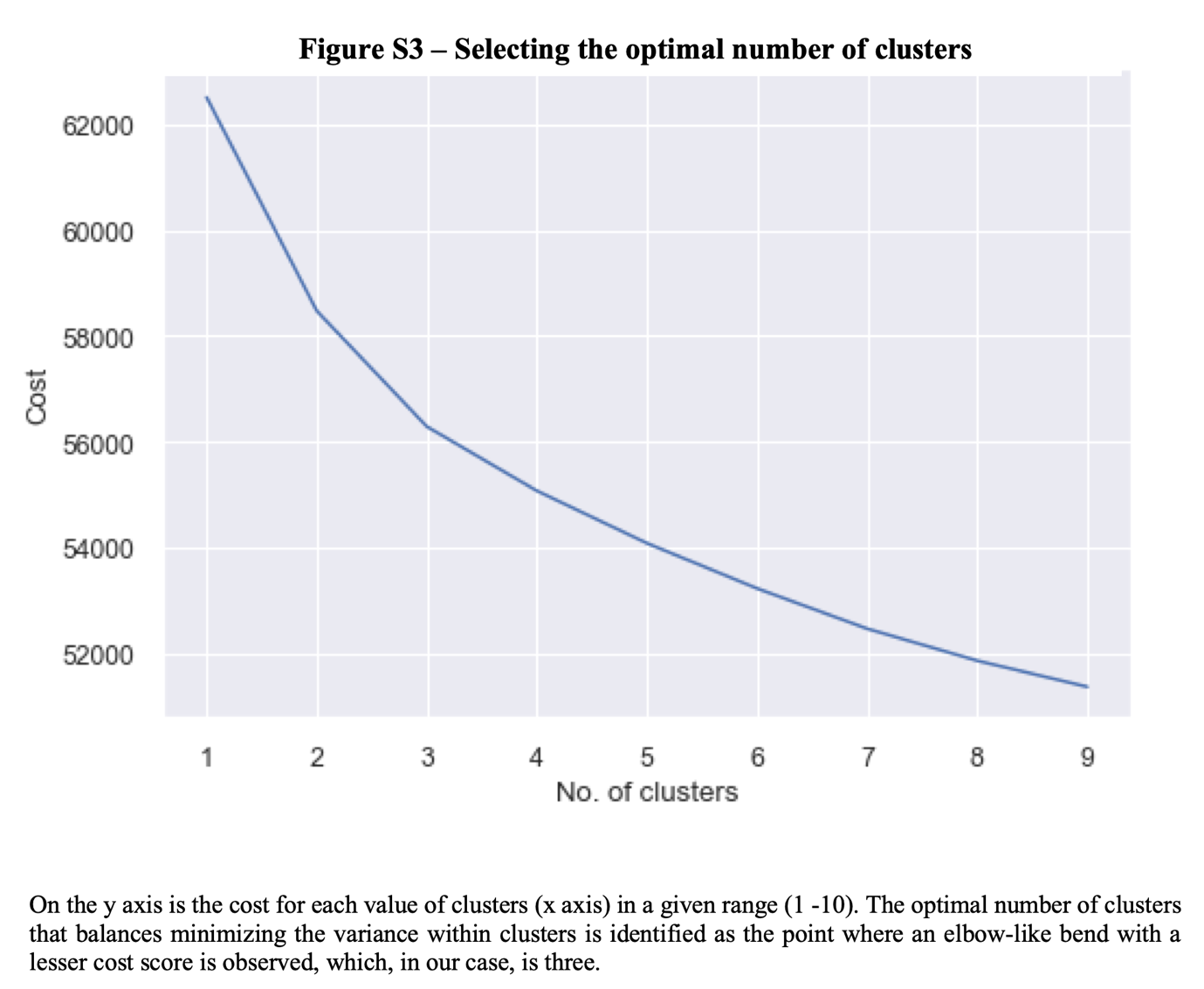


*Comparing characteristics among clusters and their associations with clinical outcomes*

The obtained three clusters were compared in clinical characteristics (phenotypic domains), and imaging features (CMR-derived metrics) using chi-squared tests for categorical variables, and analysis of variance (or Kruskal-Wallis test, when appropriate) for continuous variables. Pairwise comparisons between the clusters were performed using independent t-tests (if data were normally distributed) or Wilcoxon-rank sum tests (if data were skewed) for numeric variables, and chi-squared tests for categorical variables.

Clinical outcomes occurred after the baseline (incident events) were derived using a combination of selected UK Biobank fields with dates recorded to provide censor dates. In the survival analyses, we censored individuals according to the date the event occurred, date of death, or the end of follow-up (26 March 2021), whichever came first. Kaplan-Meier analysis was used to explore whether the cluster groups had different clinical trajectories, and the log-rank test compared the survival curves. Unadjusted and multivariate-adjusted Cox proportional hazards models were also used to assess the association between cluster membership and outcomes (incident HF, other CV, all-cause mortality, and MACE).

The proportional hazard assumptions were assessed using the *COXPHFitter.check_assumptions* method from *lifelines 0.16.0* library as described online (<https://lifelines.readthedocs.io/en/latest/jupyter_notebooks/Proportional%20hazard%20assumption.html>). Specifically, the assumptions were evaluated using statistical tests to test for any time-varying coefficients, and graphical diagnostics based on the scaled Schoenfeld residuals against the four-time transformations, and no significant violation was found.

The covariates used in the adjusted models included traditional CV risk factors (hypertension, diabetes, previous MI) and LVEF values, which are known as strong predictors of CV events and were not used as input data for clustering. Precisely, model 1 was adjusted for only CV risk factors, and model 2 was adjusted for CV risk factors plus LVEF values. Differential associations between cluster membership and LVEF with clinical outcomes were also tested using interaction terms in the Cox regression models.

A two-sided P <0.05 was considered statistically significant for all analyses. All analyses were performed using Python 3.8.10 (Python Software Foundation, Delaware USA) and Scikit-learn version 0.23.2 (23).

**Supplemental References**

1. Chadeau-Hyam M, Bodinier B, Vermeulen R, Karimi M, Zuber V, Castagné R, et al. Education, biological ageing, all-cause and cause-specific mortality and morbidity: UK biobank cohort study. EClinicalMedicine [Internet]. 2020 Dec 1 [cited 2022 Aug 31];29–30. Available from: https://pubmed.ncbi.nlm.nih.gov/33437953/

2. Franssen FME, Rutten EPA, Groenen MTJ, Vanfleteren LE, Wouters EFM, Spruit MA. New reference values for body composition by bioelectrical impedance analysis in the general population: results from the UK Biobank. J Am Med Dir Assoc [Internet]. 2014 [cited 2022 Apr 25];15(6):448.e1-448.e6. Available from: https://pubmed.ncbi.nlm.nih.gov/24755478/

3. Després JP. Body fat distribution and risk of cardiovascular disease: An update. Circulation [Internet]. 2012 Sep 4 [cited 2022 Aug 31];126(10):1301–13. Available from: https://pubmed.ncbi.nlm.nih.gov/22949540/

4. Papier K, Fensom GK, Knuppel A, Appleby PN, Tong TYN, Schmidt JA, et al. Meat consumption and risk of 25 common conditions: outcome-wide analyses in 475,000 men and women in the UK Biobank study. BMC Med [Internet]. 2021 Dec 1 [cited 2022 Aug 31];19(1). Available from: https://pubmed.ncbi.nlm.nih.gov/33648505/

5. Craig CL, Marshall AL, Sjöström M, Bauman AE, Booth ML, Ainsworth BE, et al. International physical activity questionnaire: 12-Country reliability and validity. Med Sci Sports Exerc [Internet]. 2003 Aug 1 [cited 2022 Aug 31];35(8):1381–95. Available from: https://pubmed.ncbi.nlm.nih.gov/12900694/

6. Petersen SE, Matthews PM, Francis JM, Robson MD, Zemrak F, Boubertakh R, et al. UK Biobank’s cardiovascular magnetic resonance protocol. J Cardiovasc Magn Reson [Internet]. 2016 [cited 2020 May 27]; Available from: www.ukbiobank.ac.uk

7. Petersen SE, Aung N, Sanghvi MM, Zemrak F, Fung K, Paiva JM, et al. Reference ranges for cardiac structure and function using cardiovascular magnetic resonance (CMR) in Caucasians from the UK Biobank population cohort. J Cardiovasc Magn Reson. 2017;19(1).

8. Bai W, Sinclair M, Tarroni G, Oktay O, Rajchl M, Vaillant G, et al. Automated cardiovascular magnetic resonance image analysis with fully convolutional networks 08 Information and Computing Sciences 0801 Artificial Intelligence and Image Processing. J Cardiovasc Magn Reson [Internet]. 2018 Sep 14 [cited 2021 May 28];20(1). Available from: https://pubmed.ncbi.nlm.nih.gov/30217194/

9. Cheng S, Fernandes VRS, Bluemke DA, McClelland RL, Kronmal RA, Lima JAC. Age-related left ventricular remodeling and associated risk for cardiovascular outcomes the multi-ethnic study of atherosclerosis. Circ Cardiovasc Imaging [Internet]. 2009 May [cited 2021 Dec 2];2(3):191–8. Available from: https://www.ahajournals.org/doi/abs/10.1161/circimaging.108.819938

10. Mewton N, Opdahl A, Choi EY, Almeida ALC, Kawel N, Wu CO, et al. Left ventricular global function index by magnetic resonance imaging - A novel marker for assessment of cardiac performance for the prediction of cardiovascular events: The multi-ethnic study of atherosclerosis. Hypertension [Internet]. 2013 Apr [cited 2021 Dec 2];61(4):770–8. Available from: https://www.scholars.northwestern.edu/en/publications/left-ventricular-global-function-index-by-magnetic-resonance-imag

11. Nwabuo CC, Moreira HT, Vasconcellos HD, Mewton N, Opdahl A, Ogunyankin KO, et al. Left ventricular global function index predicts incident heart failure and cardiovascular disease in young adults: The coronary artery risk development in young adults (CARDIA) study. Eur Heart J Cardiovasc Imaging. 2019;20(5):533–40.

12. Ikonomidis I, Aboyans V, Blacher J, Brodmann M, Brutsaert DL, Chirinos JA, et al. The role of ventricular–arterial coupling in cardiac disease and heart failure: assessment, clinical implications and therapeutic interventions. A consensus document of the European Society of Cardiology Working Group on Aorta & Peripheral Vascular Diseas. Eur J Heart Fail [Internet]. 2019 Apr 1 [cited 2022 May 9];21(4):402–24. Available from: https://onlinelibrary.wiley.com/doi/full/10.1002/ejhf.1436

13. Redheuil A, Wu CO, Kachenoura N, Ohyama Y, Yan RT, Bertoni AG, et al. Proximal aortic distensibility is an independent predictor of all-cause mortality and incident CV events: The MESA study. J Am Coll Cardiol [Internet]. 2014 Dec 23 [cited 2022 May 11];64(24):2619–29. Available from: /pmc/articles/PMC4273646/

14. UK Biobank Pulse wave analysis and aortic pressure. October 2015. Available at: https://biobank.ndph.ox.ac.uk/showcase/ukb/docs/vicorder_explan_doc.pdf. Accessed December 1, 2021.

15. Biasiolli L, Hann E, Lukaschuk E, Carapella V, Paiva JM, Aung N, et al. Automated localization and quality control of the aorta in cine CMR can significantly accelerate processing of the UK Biobank population data. PLoS One [Internet]. 2019 Feb 1 [cited 2021 Dec 1];14(2). Available from: https://pubmed.ncbi.nlm.nih.gov/30763349/

16. Mandry D, Girerd N, Lamiral Z, Huttin O, Filippetti L, Micard E, et al. Relationship Between Left Ventricular Ejection Fraction Variation and Systemic Vascular Resistance: A Prospective Cardiovascular Magnetic Resonance Study. Front Cardiovasc Med [Internet]. 2021 Dec 24 [cited 2022 Apr 21];8:803567. Available from: /pmc/articles/PMC8739894/

17. Chirinos JA, Rietzschel ER, De Buyzere ML, De Bacquer D, Gillebert TC, Gupta AK, et al. Arterial load and ventricular-arterial coupling: Physiologic relations with body size and effect of obesity. Hypertension [Internet]. 2009 Sep 1 [cited 2022 Jun 20];54(3):558–66. Available from: https://pubmed.ncbi.nlm.nih.gov/19581507/

18. Chirinos JA, Segers P. Noninvasive evaluation of left ventricular afterload: Part 2: Arterial pressure-flow and pressure-volume relations in humans. Hypertension [Internet]. 2010 Oct [cited 2022 Jun 20];56(4):563–70. Available from: https://pubmed.ncbi.nlm.nih.gov/20733088/

19. Huang Z. Extensions to the k-means algorithm for clustering large data sets with categorical values. Data Min Knowl Discov [Internet]. 1998 [cited 2022 Apr 20];2(3):283–304. Available from: https://link.springer.com/article/10.1023/A:1009769707641

20. Preud’homme G, Duarte K, Dalleau K, Lacomblez C, Bresso E, Smaïl-Tabbone M, et al. Head-to-head comparison of clustering methods for heterogeneous data: a simulation-driven benchmark. Sci Rep [Internet]. 2021;11(1):1–14. Available from: https://doi.org/10.1038/s41598-021-83340-8

21. Satopää V, Albrecht J, Irwin D, Raghavan B. Finding a ‘kneedle’ in a haystack: Detecting knee points in system behavior. In: Proceedings - International Conference on Distributed Computing Systems. 2011.

22. Levine E, Domany E. Resampling method for unsupervised estimation of cluster validity. Neural Comput. 2001;13(11):2573–93.

23. Pedregosa F, Varoquaux G, Gramfort A, Michel V, Thirion B, Grisel O, et al. Scikit-learn: Machine learning in Python. J Mach Learn Res. 2011;12.
